# Nanoparticles assisted intra and transdermic delivery of antifungal ointment: an updated review

**DOI:** 10.1186/s11671-023-03932-3

**Published:** 2024-01-09

**Authors:** Nazia Tarannum, Km. Pooja, Shivani Jakhar, Anshika Mavi

**Affiliations:** Department of Chemistry, Chaudhary Charan Singh University, Meerut, 250004 Uttar Pradesh India

**Keywords:** Nanoparticles, Nanotechnology, Emulsion, Drug delivery, Antifungal

## Abstract

This review paper highlights the trans-dermic delivery of nanoparticles (NPs) based antifungal ointments with the help of nanotechnology. It also describes the novel trans-dermal approach utilizing various nanoparticles which enables an efficient delivery to the target site. This current review gives an overview about past research and developments as well as the current nanoparticle-based ointments. This review also presents data regarding types, causes of infection, and different pathogens within their infection site. It also gives information about antifungal ointments with their activity and side effects of antifungal medicines. Additionally, this review also focuses on the future aspects of the topical administration of nanoparticle-based antifungal ointments. These nanoparticles can encapsulate multiple antifungal drugs as a combination therapy targeting different aspects of fungal infection. Nanoparticles can be designed in such a way that they can specifically target fungal cells and do not affect healthy cells. Nanoparticle based antifungal ointments exhibit outstanding potential to treat fungal diseases. As further research and advancements evolve in nanotechnology, we expect more development of nanoparticle-based antifungal formulations shortly. This paper discusses all the past and future applications, recent trends, and developments in the various field and also shows its bright prospective in the upcoming years.

**Graphical abstract:**

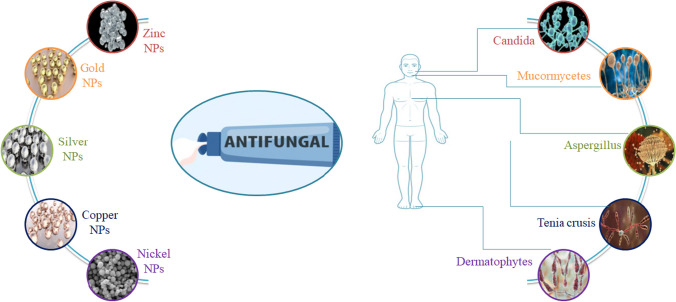

## Introduction

Numerous changes in the modern environment have brought exposure to various strains of fungi, this has made the emergence of several fungi-related problems. These problems or the extent of infection caused by them depend upon the degree of interaction between the infectant and the area of the organism being infected [1]. Around 3 to 6 million fungal species have been reported globally from which 200 to 800 species are mainly involved in causing diseases in the human race [2]. The attack of pathogens is a two-way mechanism, first survival, and growth of microorganisms on the host, second damaging the host and disrupting homeostasis to show disease symptoms [3]. Around 1/4th of the human race present in the environment suffers from superficial infection of the dermal layer [4]. In people with weakened immune systems, infections are more likely to be brought by fungus. An infection caused by fungus is susceptible anywhere on the body. Some of the most prevalent are Onychomycosis, Candidacies, etc. Some fungal infections are associated with high mortality (chance of death) rates [5]. The most common fungi that cause serious or life-threatening infection is Aspergillus which causes aspergillosis [6]. It most often affects people with lung disease or weakened immune system.

**Fig. 1:**
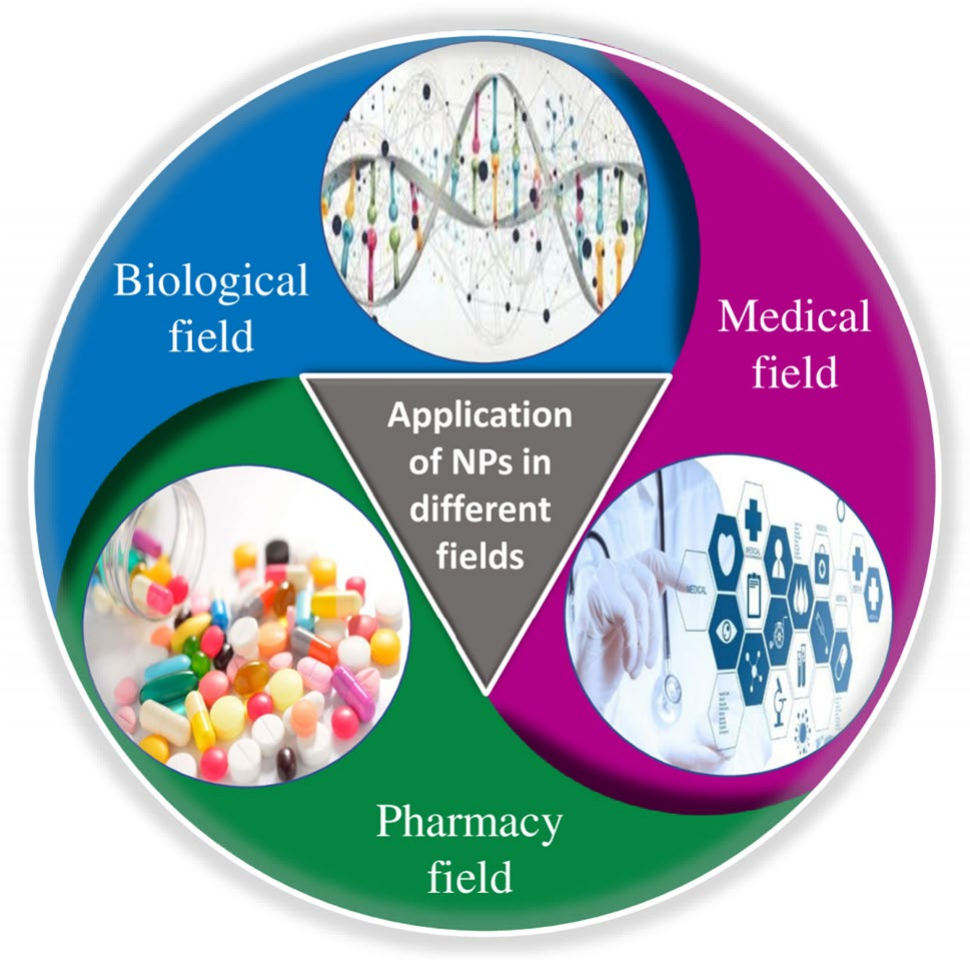
Application of NPs in different fields

In today’s world, several antifungal agents are being manufactured and used to prevent and treat further infection and growth. But the search for more efficient and easier applications is being done to find a way towards enhanced therapy and efficiency of antifungal ointment. The effectiveness of antifungal medications used to treat fungi-based infections depends on several characteristics, including solubility, penetration through tissue, drug stability, etc. Although there are numerous antifungal agents available, the search for fast-absorbing ointment & other drug delivery options are always a point of research [7]. The rapid absorption and transmission to a certain depth of skin is primarily dependent upon the nature of the drug transmitter [8, 9]. The use of nanomedicine and nanotechnology has been increased recently to overcome the demerits of other drug transmitters in preventing diseases caused by microbes and other injuries. The three major concerned areas focused under nanoparticles and nanomedicines are nano-instruments and nanomaterials in biological fields, pharmacy fields, and medical fields as represented in Fig. 1 [10]. There are some most frequently used applications of NPs in biological fields such as hyperthermia-based tumordestruction, brightly colored biological labels, delivery of drugs and genes, pathogenbio-detection, proteindetection, DNA structure investigation, engineering of tissue, cell, separation and purification of biological molecule, MRI contrast augmentation, studying phagokinetic [11]. Nanomaterials can be used for bio tagging or biolabeling since they fall within the same size range as proteins (nanoparticles). For the purpose of employing nanoparticles as biological indicators, size is only a single characteristic out of numerous characteristics of nanoparticles that is rarely sufficient as a whole itself [12].

Salata et al. were the first to reveal the potential of the controlled release of macromolecule drugs through the use of polymer leading to the advancement of the drug release for photodynamic cancer therapy [13, 14]. The general size of nanoparticles is in the range of 1–100 nm [15]. Several uses where NPs are being broadly used are—tissue engineering scaffolds, target drug delivery, and major or minor disease diagnosis [16, 17]. For good NPs to be used for the pharmaceutical purpose it should possess biocompatibility and bio-degradability as their important properties. Along with the ease of production the optimum mechanical properties and the release time are also some properties of NPs taken into consideration while synthesis or production NPs based ointments [18, 19].

Different varieties for NPs may depend upon the methods of preparation of those NPs along with some other factors such as drug loading capacity, drug delivery period, shelf period, and many other [20].

## Past research and discussion

One of the main issues in the pharmaceutical and cosmetic industries is cutaneous infections [21]. By 2021, it is anticipated that the global market for skin disease treatment technologies would have grown to $20.4 billion from its estimated $17.1 billion in 2015, and revenue is expected to show an annual growth rate (CAGR 2023–2028) of 5.62%, resulting in a market volume of US$21.65bn by 2028. Innovative treatments for specific skin diseases have gained interest in the industry among big pharma corporations. To treat diverse skin disorders and infections, new dermatological and cosmetic formulations with antibacterial and antifungal properties are used. H. Linda Jeeva Kumari disclosed that ZnO is likewise regarded as one of the GRAS (generally acknowledged as safe) elements for people [22]. It blocks bacterial enzymes such as thiol peroxidases, glutathione reductases, and dehydrogenases as a result of its powerful antibacterial activities [23]. ZnO has an antifungal effect by causing fungal hyphae to deform and by preventing the growth of conidiophores, which causes the fungal hyphae to die and cause cellular damage [24]. Antibiotic resistance and rise and reemergence of infectious diseases caused by microorganisms have both been concerning trends for global health. The rise of pathogens that are multi-drug resistant (MDR) and its effects on the global economy and preventive healthcare system are of increasing concern [25]. The World Health Organization (WHO) has ranked MDR pathogens as one of the top three risks to public health.

Bukola Christiana Adebayo-Tayo and Samuel Oluwa Dara boredly reveal that Emerging technology known as nanotechnology deals with structures with a minimum of one dimension and a size of 1–100 nm. Nanotechnology is increasingly being used for antibacterial purposes, and metal nanoparticles (NPs) are increasingly being used in place of antibiotics [26]. By using materials at the nanoscale, the combination of biosynthesis and nanoparticles opens up new possibilities for the control and prevention of infectious pathogens [27]. Interest of scientist in the green chemistry method has led to its active inclusion in current nanobiotechnological research. A noble metal, silver has special qualities such as strong antibacterial effectiveness, catalytic activity, and chemical stability [28].

It is largely used in water, agriculture, textile and many biological domains. Ionic, nanoscale, and bulk metallic forms of silver are all used as antimicrobials [29]. Silver nanoparticles have been regarded as crucial metal nanoparticles with good properties and a variety of uses in a variety of industries and the creation of new goods. Pathogens with multiple drug resistanceand re-emergence may be solved through research into nanosilver [30]. A fungus that only affects the topmost layers of the skin, such as *Pityriasis versicolor, Tineanigra*, and black/white Piedra is the main cause of superficial mycoses. Fungal infections that affect thehair and nails are examples of the skin's extremities, are known as cutaneous mycoses or dermatomycoses. When they penetrate further into the epidermis contrary to superficial mycoses, cutaneous mycoses can cause pathological changes by inducing cellular immune responses [31]. Dermatophytes, often known as the fungi that cause cutaneous mycoses, typically come from the genera Microspore, Trichophyton, and Epidermophyton. The fungi-related disorders (known as dermatomycoses) include *Tinea corporis*, *Tinea pedis*. The most common form of dermatophytosis is tumefacien, tiemonium, tineacruris, *Tineabarbae*, and tineacapitis [32, 33]. Sporothrix Schenckii the fungus that most frequently causes sporotrichosis, a kind of subcutaneous infection. In areas exposed to fungal inoculation, sporotrichosis is characterized by infiltrating nodular or ulcerated lesions [34]. Chromomycosis and maduramycosis (or mycetoma) are two other instances of subcutaneous mycoses [35].

According to Eman Ahmed Bseiso, the necessity for the creation of new medications increases throughout the time. Even though they are severe enough skin fungus infections are typically treated with conventional delivery methods including lotions, ointments, and gels. The instantaneous release of therapeutics can be mitigated through transporter systems. Through the properties of these traditional compositions, they are able to prevent the danger of triggering allergic reactions [36]. Topical skin delivery methods have drawn a lot of interest in this context because of the numerous benefits associated with this mode of administration. Topical medication delivery methods employ a variety of nanocarrier types. The most widely used nanocarriers for topical drug administration include dendrimers, ectosomes, transferases, liposomes, and niosomes, which are lipid-based nanoparticles [37, 38]. Due to more stability of polymeric NPs than lipid-based systems, polymeric NPs are most widely used of all NPs [39]. The well-known antimycotic, fluconazole (FLZ) has a wide range of physic-chemical characteristics and a high bio-availability. Commercially speaking, FLZ is offered as oral tablets, capsules, and intravenous solutions. When compared to other azoles like miconazole, itraconazole, and ketoconazole, FLZ has antimycotic activity and is significantly more hydrophilic (8 mg/ml at 37 C) [40]. Due to the inclusion of one halogenated phenyl ring and two triazole rings, it also has a high bioavailability. The investigations by Aimen Khalid and Asim Naved Ahmad proved that the FLZ-NPs-loaded ointment could be a barrier to topical shipment [41].

## Cause of fungal infection and its symptoms

Numerous fungi that are part of our everyday environment might cause fungal diseases. Most individuals can regularly come into contact with fungi without experiencing any negative effects; however, under specific circumstances the fungi can overgrow and produce symptoms [42].

### Fungi

**Fig. 2:**
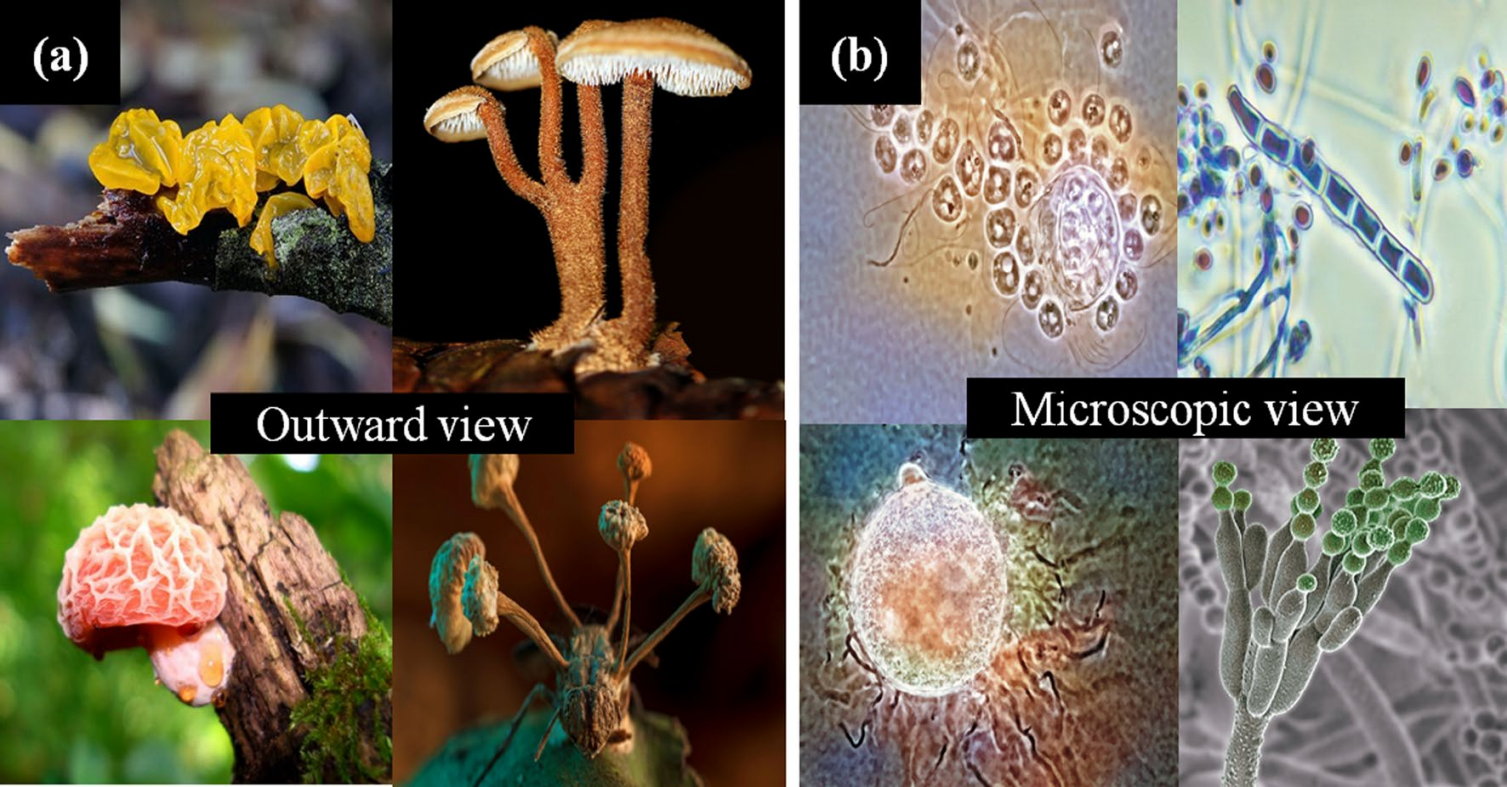
Representation of the outward and microscopic view of fungi

Fungi are eukaryotic, which means that they have a membrane covering their nucleus [43]. They disperse or expel spores into the environment to move numerous fungi naturally live in our bodies in the mouth, gastro-intestinal pathways, and skin, yet they are capable of expanding out of regulation as shown in Fig. 2a which represents the outward view of fungi. It is a non-motile living form, and the basic structural unit can either be a single cell, as seen in Fig. 2b, a chain of cylindrical cells (hyphae), or both [170].

**Fig. 3:**
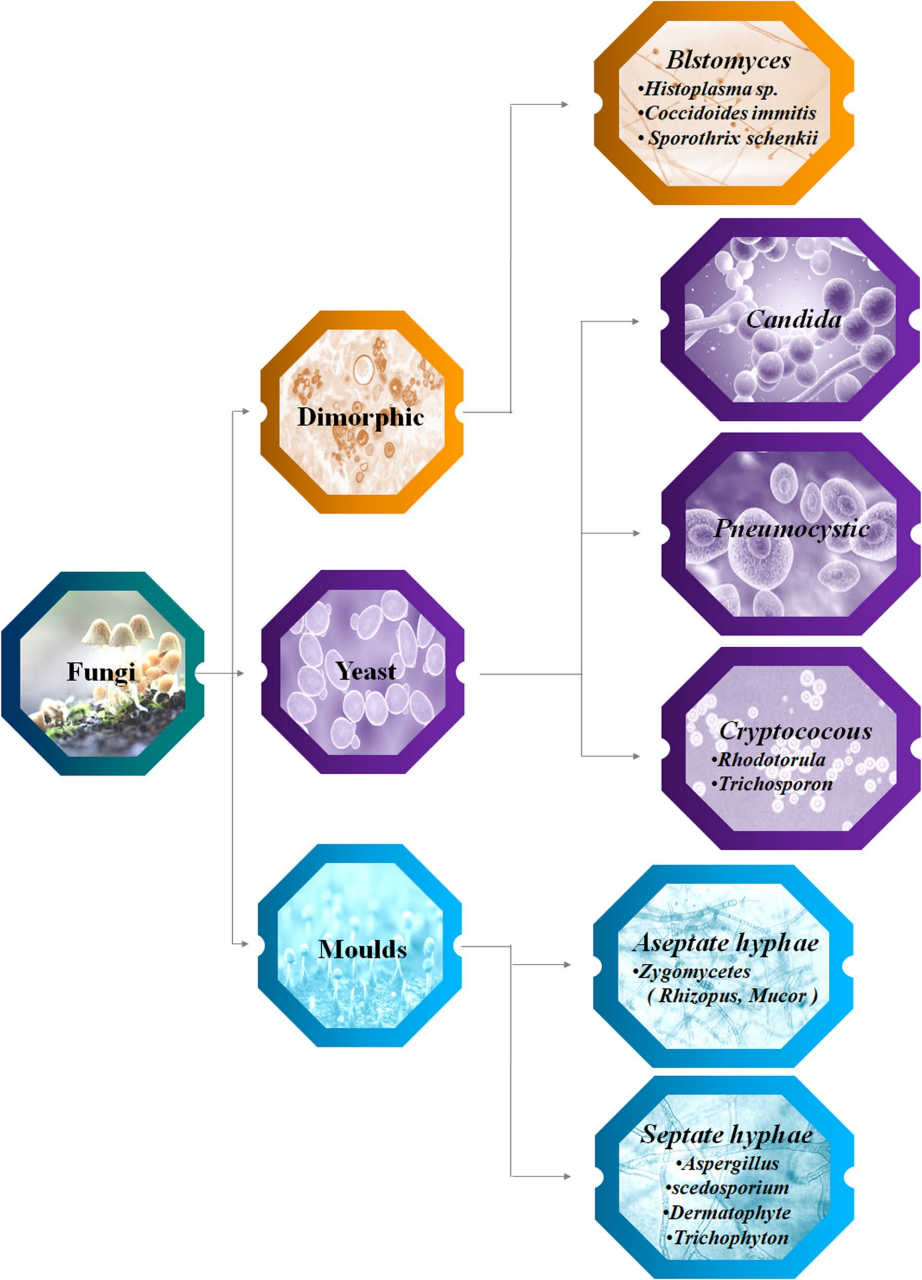
Representation of classification of fungi

Despite the fact that there exist about 5.1 millions of fungi [44] on the planet, just a few percent of them have been determined to be toxic to humans. The invasive fungal infection must be listed among the illness that is often opportunistic [45]. There are the most common species like Aspergillus and candida are found everywhere and has the ability to cause deadly infection. Recently, it has been proposed that 12–10 product failure, toll-like receptor, polymorphism, plasminogen polymorph, and other genetic variationswithin important innate or adaptive immune response genes increase immunity to invasion fungi [46]. Pathogens have undergone a clear evolution over the past few decades [47]. Even with fungi with built-in virulence may penetrate the body and cause serious disease. Figure 3 represent the classification of fungi.

### Fungal infection

A fungus, yeast, or mold causes a condition known as mycosis, sometimes known as fungal infection. Such fungi can infect the mouth, throat, lungs, urinary system, and many other regions of the human body which are known by different types of names [48]. Table 1 represents fungi and its infection along with the site of infection and symptoms.

**Table 1:** Fungi and infection along with the site of infection and symptoms

S. no.	Pathogenic fungi	Infection	Site of infection	Symptoms
1	*Candida*	Candidiasis	Skin, mucous membrane	Rashes, redness, intense itching
2	*Dermatophyte* (*Tinea ungunium*)	Onychomycosis	Nails	Decoloring of nails, thickening or brittle
3	*Tineacapitis*/*Tineamannum*	Dermatophytosis (Ringworm)	Feet, Groins, Thigh, Scap, Hands, etc.	Darkening of skin, peeling, red rashes, hair loss and itchy scalp
4	*Malassizia*	Tinea vericolor/Pityricolor	Skin (skin chol)	Decoloring of skin darkening or redness
5	*Candida albicans*	Vulvoginitis	Vagina (PRT)	Abnormal vaginal discharge, vaginal bleeding, vaginal irritation, vaginal itching, vaginal odor
6	*Coccidioides*	Coccidioides mycosis	Skin	Drowsiness (tiredness), coughing, fever, shortness of breath, headache, joint pain or pains in the muscles rash on the legs or upper body
7	*Aspergillus*	Aspergillosis	Lungs	High fever, heartache, cough, spitting blood, breathing difficulty
8	*Rhizopus*, *Mucor*	Mucormycosis	Lungs, Central nervous system	Face edema on one side headache, nasal congestion, sinus congestion rapid worsening of black lesions on the bridge of the nose or the upper interior of the mouth
9	*Trichophyton rubrum*	TineaCruris	On the thighs, on the genitals	Itchy skin, scaly patches, and a rash bordered by tiny blisters
10	*Mucormycetes*	Zygomycosis	Brain and nasal sinuses	Fever, headache, coughing, shortness of breath, abdominal pain, bloody vomit
11	*Histoplasmacapsulatum*;	Histoplasmosis	Lungs, skin	Chest pain, a dry cough, headaches, muscle aches, fatigue, and fever
12	*Cryptococcus gattii*	*C. gattii* cryptococcosis	Lungs, Central Nervous System	Cough, shortness of breath, chest pain, fever
13	*Sporothrix*	Sporotrichosis	Skin	Small pink, red or purple painless bump resembling an insect bite
14	*Paracoccidioides*	Para coccidioidomycosis	Lungs, skin	Lesion in the mouth and throat, weight loss, swollen lymph nodes, cough, fever, shortness of breath, fatigue, enlarged liver and spleen
15	*Talaromycesmarneffei*	Talaromycosis	Whole body	Fever, unease, decreased appetite, cough, diarrhoea, and respiratory problems

### How does a fungal infection appear?

Fungi caused skin infections may be rough, red or bloated, you might be able to feel a lump under your skin, or they might look like a rash. Fungi-caused infections may also cause your nail to thicken, become brittle, or change color (to yellow, brown, or white). White patches or coatings might appear when the mouth or throat is infected with fungus [49].

### Different types of fungal infection

**Fig. 4:**
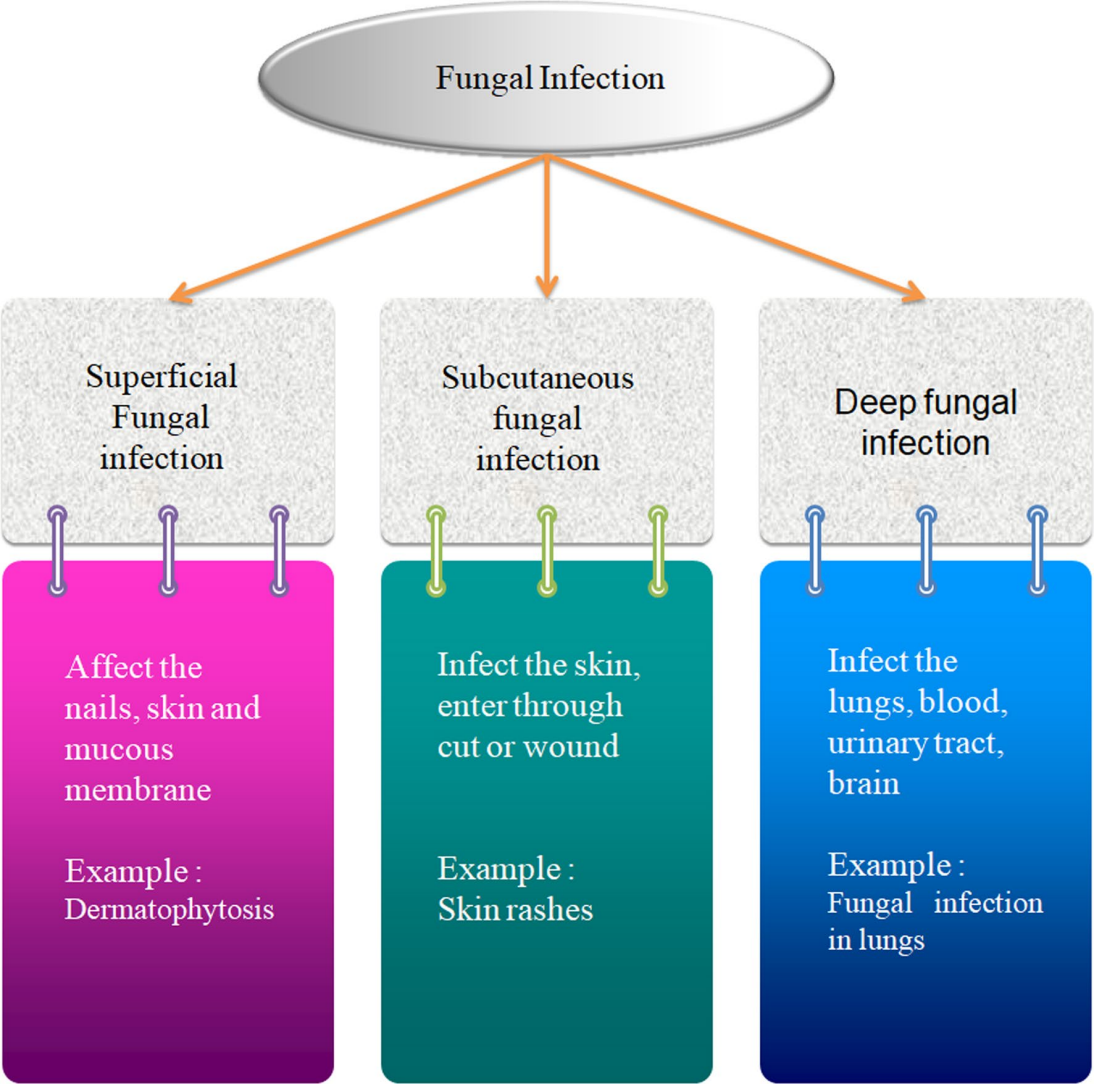
Representation of flow chart of fungal infection

Fungal infections of the skin can have rough, swollen, or red appearances [50]. They may appear as a rash or as a lump that you can see under your skin. Figure 4 shows a different types of fungus infections based on their symptoms in the human body such as our nails becoming thick, brittle, or discolored (yellow, brown, or white), Infections with fungi in the mouth or throat may cause a white coating or patches, etc. An infection by a fungus can affect our skin, nails, or mucous membranes on the exterior of our bodies (superficial or mucocutaneous infection), beneath our skin (subcutaneous infection), or inside an organ like the lungs, brain, or other tissues.

#### Superficial fungal infection

The superficial fungal infection affects your nails, skin and mucous membrane (like mouth, throat, and vagina). Examples-Dermatophytosis, onychomycosis, candidiasis, and tinea versicolor [51].

#### Subcutaneous fungal infection

It is the fungal infection beneath the skin, if the fungus entersintoa cut or wound, generally as a result of an injury sustained while working with a plant (such as a scratch from a thorn). This may develop a fungal infection beneath the surface of the skin. Examples-skin rashes, ulcers, etc. [52]. Tropical and subtropical regions of the world have a higher prevalence of subcutaneous fungal infection. Examples of subcutaneous fungal infection are sporotrichosis and chromo blastomycosis.

#### Deep fungal infection

Deep fungal infection is formed in places like—lungs, blood, urinary tract, brain, etc. Some are only opportunistic infections, i.e., they usually cause disease in people with weekend immune systems. Deep or invasive fungal infections include—*Histoplasmosis, Histoplasma* in plasmolysis, the fungus can infect one’s lungs, brain, etc. commonly found in the Ohio and Mississippi River valley [53].

## Antifungal and antimycotic ointment and their fungicidal activity

There are numerous types of antifungal drugs. Injections, shampoos, pessary tablets (tablets that are meant to be placed into the vagina), lotions, sprays, solutions, and other forms are also available [54]. Most act by breaking down the fungus cell wall, which kills the fungal cell. Antifungal ointment clears the infection whereas mild steroid ointment reduce the inflammation caused by the infection. A fungicide or fungi static medication used to treat and Prevent mycosis is an antifungal medication, often known as an antimitotic medication. The most popular formulation for treating numerous dermatological diseases is ointment.

### Antifungal medication types

**Fig. 5:**
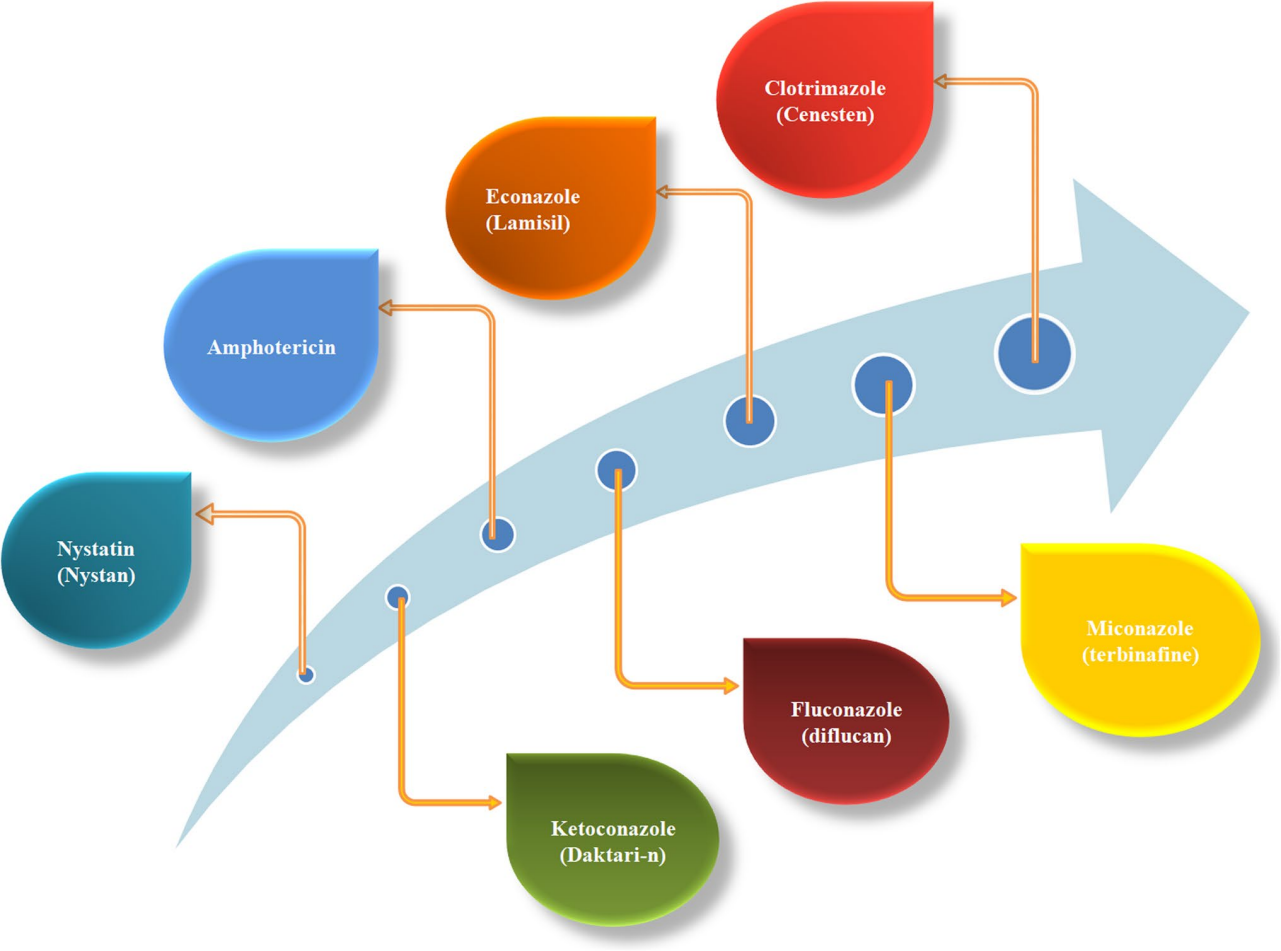
Different antifungal medication

Antifungal agents can be used in Ointments, gels or sprays, pills, capsules, liquid or injections, and medicines. In general, ointments are commonly used to treat fungal infections [55]. The reason antifungal ointments are preferred over pills, capsules, and injections are that young children should not take antifungal tablets but should accept used antifungal ointments and many others. Commonly mentioned active pharmaceuticles ingredients in antifungal ointment such as clotrimazole, econazole, miconazole, fluconazole, and ketoconazole amphotericinas represented in Fig. 5 and Table 2.

**Table 2:** Describe the formulation and function of azole antifungal agents

S. N.	Antifungal agent (API)	Formulation	Used to treat	Function	Side effect	References
01	Miconazole	Lotion	Fungal infection	By stop the growth of fungus	Itching, irritation, redness and swelling	[56]
02	Fluconazole	Oral suspension and tablet form	Yeast and fungal infection	Stop the growth of fungus	Vomiting, stomach pain	[57]
03	Verniconazole	Cream	Fungal infection	Stop the growth of fungus	Pain, redness	[58]
04	Ketoconazole	Oral tablet, cream and shampoo	Fungal infection	By destroy the cell wall of fungus	Skin rashes, irritation and redness	[59]
05	Clotrimazole	Oral, topical administration ointment formulation, and vaginal cream	Fungal and microbial infection	Used to treat skin infection by prevent the growth of fungus	Itching rashes	[60]
06	Econazole	Cream	Fungal and yeast infection	Stop the growth of infection	Swelling, skin rashes	[61]

### Side effects of antifungal medicine

Antifungal drugs can cause a variety of side effects. They are usually short-lived and are of moderate severity [62]. These may include redness or itching, abdominal discomfort, vomiting, and rashes. Antifungal prescription drugs may occasionally result in more severe side effects, including severe skin reactions that result in blistering or peeling of the skin, very rare liver damage, fatigue or weakness, lack of appetite, nausea, vomiting, jaundice, pale urine or faeces, or an amalgamation of these symptoms [54].

### How antifungal drugs function?

Antifungal medication can kill the fungus cell wall or stopping them it from growing and multiplying. Antifungal ointment mainly target the cell wall of the fungus. Antifungal medications can get rid of the fungus or prevent it from growing. To cure infections caused by fungi of the layers of skin, scalp conditions and nails, antifungal ointments, liquids, and sprays are employed [63]. These are some important salts used in antifungal ointments—clotrimazole, terbinafine, amorolfine, miconazole, ketoconazole, miconazole, and tioconazole. They can be found in an extensive range of manufacturers. An antifungal ointment can occasionally be coupled with other ointments when two actions are required. For instance, a moderate steroid ointment like hydrocortisone is frequently used in conjunction with an antifungal ointment to treat a particular rash. Antifungal ointment aids in the removal of the infection while moderate steroid ointment reduces inflammation brought on by infection. The broad-spectrum antifungal is a typical treatment for invasive fungal infections [58].

## Nanoparticles (NPs) as a crucial component in antifungal ointment

**Fig. 6:**
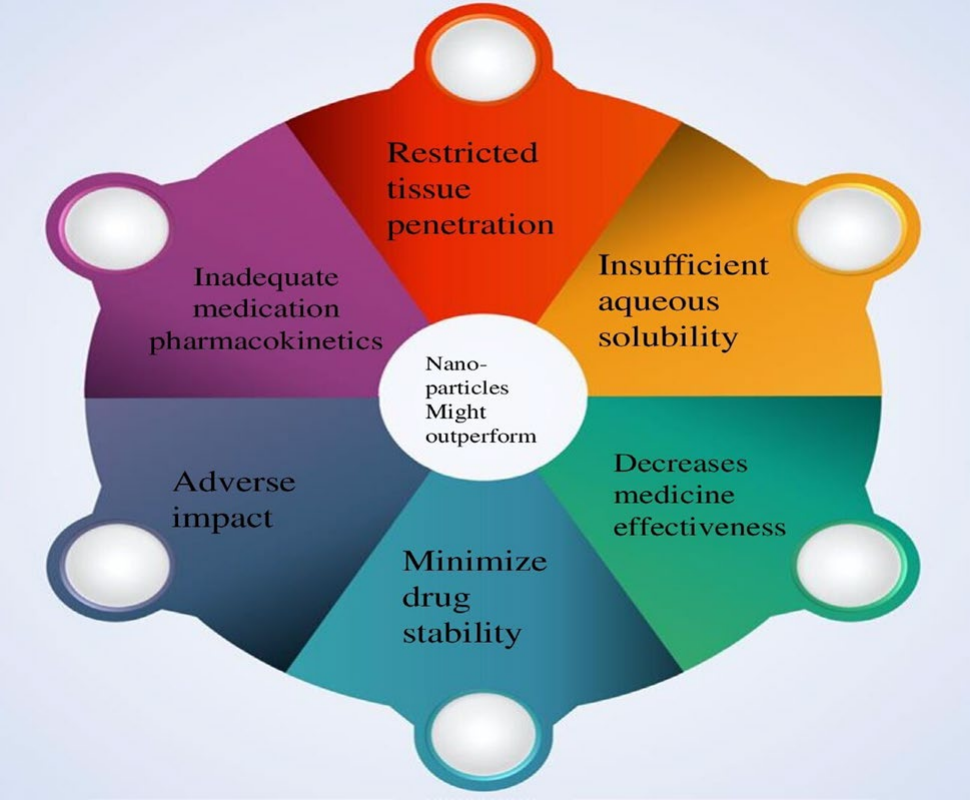
Demerits of antifungal ointment that overcome by nanoparticles

Nanoparticles hold considerable promise for overcoming these constraints because of their capacity to improve medication bioavailability, solubility in water, and antifungal activity [64]. Additionally, incorporating drugs into NPs could significantly lessen their toxicity. There are not many commercialized NPs based antifungal medication compositions despite these intriguing NPs characteristics. This study discusses different types of NPs, including nanoemulsions, solid lipid NPs, polymeric micelles, nanostructured lipid carriers, lipid-based vesicles, and dendrimers, used in antifungal drug delivery [65]. Focusing research on resolving issues with NPs stability, drug loading capacity, high costs production, and standardization could make it easier to get these nanoformulations to the clinic from the lab. There are some demerits of general antifungal ointments (Fig. 6) like-poor aqueous solubility, low drug efficacy, low drug stability, poor drug pharmacokinetics, restricted penetration through tissue, and other various side effects.

## Different type of anti-fungal ointment based on nanoparticles (NPs)

In this study, we featured past work that has applied nanotechnology to convey antifungal treatments. The controlled and sustained release of antifungal ointments in a variety of routes of administration—oral, topical, ocular, pulmonary, and parenteral was made possible by these systems allowing for increased penetration and deposition of the ointments in the target tissues. As a result, using nanotechnology to deliver antifungal ointments becomes a safer and more effective treatment option for fungal infections [66]. Several azole antifungal medications, for instance, have limited formulation options and efficacy due to poor aqueous solubility, while numerous other medications have limited benefits due to toxicity. To improve NPs can significantly reduce their harmful effects. Despite these fascinating NPs hold incredible guarantees to conquer these restrictions because of their capacity nanoparticles drug water solubility, bioavailability, and antifungal viability. The advantages and disadvantages of different types of antifungal delivery methods for NPs drug, such as lipid-based vesicles, polymeric micelles, solid lipid nanoparticles, nanostructured material lipid carriers, and nano & dendritic emulsions, are the main emphasis of this review.

### Different nanoparticles (NPs) and their synthesis

This table focuses on the synthesis of NPs and their antimitotic activity. Synthesis methods including precipitation, solvothermal reduction method, chemical reduction, physiochemical processes, microemulsion hydrothermal synthesis, and refluxing method. Table 3 represents NPs and its antimitotic activity and their synthesis.

**Table 3:** Nanoparticles and antimitotic activity

S.N.	Nanoparticles	Particle size (in nm)	Zeta potential (in mv)	Poly-dispersion index	Antimitotic activity	Methods of synthesis	References
01	AgNPs (silver nanoparticles)	40–180	− 62	0.50	Prohibit fungus growth	Several irradiation techniques such as laser irradiation can be used to create silver NPs through aq. silver salt and surfactant	[67–69]
02	ZrO_2_ (zirconium oxide nanoparticles)	100	+ 48	0.09	Growth reduction	Zirconium NPs are synthesized via the coprecipitation method and hydrothermal method	[70]
03	Bismuth nanoparticles	40–60		0.456	Growth inhibitor, biofilm formation inhibitor	Synthesis of bismuth NPs can be possible through the solvothermal reduction method. Ethylene glycol is used as a solvent and reducing agent in the reduction of Bi3+	[71, 72]
04	ZnO (zinc oxide nanoparticles)	30–150	28.8 and 26.3	0.412 ± 0.039	Inhibit the action of the fungus	ZnO NPs can be produced by refluxing methods, zinc acetate dehydrates in diethylene glycol, and Tri ethylene glycol used as a precursor	[73, 74]
05	Au_2_O_3_ (gold oxide nanoparticles)	5–400	− 30	3.3 ± 0.9	Great fungicidal against candida species	Gold NPs are produced by physiochemical processes	[75]
06	NiO (nickel oxide nanoparticles)	19.9–60.4	− 6.05 to 17.9		–	NiO NPs are produced via the reaction of NiCl with hydrazine and the thermal decomposition of precursor Ni(OH)_2_ leads to produce NPs	[76]
07	Trimetallic nanoparticles (TNPs) of Au/Ag/Cu	4–10			–	The chemical synthesis approach can be used to make TNPs. The catalytic activity is increased by L-ascorbic acid and NaBH4	[77]
08	Nitric oxide		− 35.9		Growth inhibitor	A mixture of (TMOS) polyethylene glycol, glucose, and chitosan, NaNO_3_ in 0. 5 M NaPO_4_ buffer (pH 7)	[78]
09	Nanoemulsion NB-201			0.08–0.7	Growth inhibitor	Nanoemulsions can be prepared by both high and low-energy methods including high-pressure homogenization, micro-liquification, and ultrasound	[79, 80]
10	Copper nanocomposite with SiO_2_	86	− 20.6 ± 4.74	0.314	Activity against fungi like Candida	3. 65 g of sodium oleate and 1. 08 g of copper chloride were dissolved in a mixed solution of hexane, ethanol, and water that had been distilled	[81, 82]

### Uses of medical drugs on fungus and their carrier

Medical drugs are commonly used to treat fungal infections in both humans and animals. These drugs are designed to target the specific mechanisms or structures within the fungi to inhibit their growth and ultimately eliminate the infection. Here are some examples of medical drugs used to treat fungal infections and their applications. (Table 4).

**Table 4:** Medical drugs for different fungal infections

S. N.	Fungus	Medical drug	Entrapment efficiency (in %)	Drug carrier	Use	References
01	*C. albicicans*	Terbinafine hydrochloride	Above 90	Solid lipid NPs	Onychomycosis	[24]
02	*C. albicicans*	Bifonozole	95.99%	Solid lipid NPs	Antifungal agent	[83]
03	*Aspergillusniger*	Gold	–	NPs	Antifungal agent	[84]
04	*niger* + *Penicillium*	Zinc	–	NPs	Antifungal agent	[85]
05	*A. niger*	Clove essential oil	–	NPs	Fungicide	[86]
06	*Aspergillus, Candida, Fusarium, Penicillium*	Hassalidins	45.50 ± 2.34 to 92.73 ± 0.33%	NPs	Antifungal agent	[87]
07	*C. albicicans*	Itraconazole	78.56% for F_6_	NPs	Antifungal agent	[88]
08	*C. albicicans*	LL-37 Peptide + Ceragin CSA-13	–	NPs	Antifungal agent	[77]
09	*Candidiasis* + *Cryptococcsis*	Miltefosine	73.7 ± 3.7%	NPs	Antifungal	[89]
10	*C. albicicans*	Miconazole + farnesol	90.2	Polymeric NPs	Vulvovaginal candidiasis	[90]
11	*Candidiasis*species	Nystatin	45.80 ± 2.34 to 92.73 ± 0.33%	Polymeric NPs	Oral cavity	[91]
12	*C. albicicans*	Amphotericin-B	95%	PLGA NPs	Antifungal	[92]
13	*C. albicicans*	Histatin 5	–	Liposome	Oral *Candidiasis*	[93]
14	*C. albicicans*	Oxiconazole nitrate	76.05–94.64%	Ethosomagell	Contagious diseases	[94]
15	*C. albicicans*	Fluconazole	97.66 and 93.37%	Nisosomal gel	Corneal fungal infections	[95]
16	*C. albicicans*	Ketoconazole	85.12–96.5%	Liposome	Fungal infection	[96]
17	*C. albicicans*	Itraconazole	–	Spanlastics	Ocular fungal infection	[88]
18	*Aspergillus*	Amphotericin-B	–	Liposome	Burn patients	[97]

*C.* represents *Candida*

## Barrier in skin and nanoparticles (NPs) as delivery agents

The skin functions as a natural barrier, enabling the delivery of drugs through the skin. A variety of techniques have been developed in order to enhance the penetration of therapeutic properties from which one option is to use the delivery system for nanoparticulate [98]. Since in the development of liposomes and other vesicular systems, a wide variety of nanosized carriers for drugs, including lipid solid NPs, nanostructured materials lipid transporters, polymer-based NPs, and magnetic NPs for dermatological applications, have been generated [99]. This review article explores the interactions and skin barrier penetration of several particle systems. The Skin serves as a barrier to shield the body from outside effects having mechanical, chemical, microbiological, and physical effects. Due to its extensive surface area and accessibility, skin delivery bears potential for use in the delivery of therapeutics. Topical delivery is the main emphasis of skin delivery. The skin can be accessed by micro and macromolecules via three pathways are: (1) the transcellular pathway, (2) the trans appendageal route, and (3) keratinocytes. The intercellular pathway travels across the lipid matrix found in the keratinocytes' intercellular gaps.

One of the most significant and well-researched strategies to boost drug distribution via skin is the generation of structural changes beneath the skin by the application of chemical enhancers such as phospholipids, fatty acids, esters, surfactants, alcohols, polyalcohol, pyrrolidines, amines, amides, sulphoxides, terpenes, and alcohols [100]. Chemical enhancers work in a variety of ways, including (a) disrupting the highly ordered structure of SC, (b) extracting keratin and/or lipid components from stratum corneum (SC), (c) fluidizing the crystalline structure of SC, and (d) simply hydrating skin through occlusion, and (e) improving drug, co-enhancer, or solvent partitioning into SC. Lipids, sugars, polymers that degrade or don't degrade, metals, and organic or inorganic substances can all be found in nanoparticles. In comparison to chemical penetration enhancers, nanoparticles have a number of benefits, including continuous medication release over an extended length of time and preservation of encapsulated compounds against chemical deterioration. The carrier must release the encapsulated medicine so that it may be absorbed via the skin layers and disease-related subcutaneous tissues for the best topical drug administration into the skin [101].

## Current nanoparticles (NPs) used in antifungal ointments

### Clinical trials and patents

This study covers a few clinical trials that utilize nanoparticles like AgNPs and MgONPs to exhibit antifungal properties. According to the trials' findings, these nanoparticles showed encouraging antifungal effectiveness against every fungal strain that was examined [102]. They all have the ability to inhibit the growth of various fungi such as *C. albicans*, *A. fumigatus*, *C. neoformans* respectively at different concentrations. Moreover, MIC ranges of some nanoparticles against *C. albicans*, *A. fumigatus*, *B. sutflis*, *A. flavus*, *Trichophyton rubrum* were determined in the given trials that are illustrated in Table 5 [103]. There are numerous investigations which demonstrated the antifungal characteristics of NPs even at low concentrations i.e., the fungus has been reported to be inhibited by inhibiting growth and influencing the creation of mature biofilms of fungus. The endurance of NPs of preventing fungal growth is become more effective when they are associated with antifungals such as fluconazole, Miconazole, Ketoconazole, Itraconazole, Nystatin etc. Their inhibitory capacity is increased. This combination of NPs and antifungals also significant in eliminating the secondary and undesirable impacts of these two components. These results are significant for evaluating the therapeutic application of these materials [104].

**Table 5:** Represent the clinical trial of Nanoparticles in antifungal drug delivery

S.N.	Nanoparticles	Drugs	Fungi	MIC (in μg/mL)	References
1	Ag		*C. albicans*	0.500	[105]
2	MgO	Nystatin	*C. albicans*	–	
3	Ag	FLZ	*A. fumigatus*	1.000	[105]
4	Ag	FLZ	*C. neoformans*	–	
5	MgO	Ofloxacin	*B. sutflis*	–	
6	Ag	FLZ	*A. flavus*	2.000	[110
7	Ag	ITZ	*C. ablicans*	0.125	[106]
8	Ag	ITZ	*C. glabrata*	0.5	[107]
9	Ag	ITZ	*Trichophyton rubrum*	≤ 0.5	[106]
10	Ag	NYT, AMP	*C. ablicans*	0.331, 0.125	[107]
11	Ag	NYT, AMP	*C. glabrata*	0.663, 0.25	[108]
12	Ag	NYT, AMP	*C. kruzei*	2.64, 2.00	[108]
13	Ag	FLZ	*C. ablicans*	0.002	[109]
14	Ag	FLZ	*C. glabrata*	0.004	[107]

In past year, many researches have been performed in the field of nanoparticles that have been useful in areas such as medicine and molecular biology research. In particularly they may be used as antifungal agent against the fungi. There are some patents related to antifungal activities of nanoparticles often cover innovative methods or formulations utilising nanotechnology to combat fungal infections as represented in Table 6. These patents explore the use of nanoparticles to create more effective, targeted, and potential less toxic antifungal treatments. They might involve nanoparticles of various materials (such as silver, chitosan, zinc oxide, etc.) known for their antifungal properties and their applications in medical, agricultural, industrial settings. The patents generally detailed the unique compositions, methods, or applications that leverage nanotechnology for enhanced antifungal effects. Additionally, surface-modified nanoparticles with functional groups or coatings enhance their specificity toward fungal cells, minimizing adverse effects on host cells. These patents delve into complex areas, including controlled drug release systems, employing stimuli-responsive nanoparticles for targeted delivery. Encapsulation techniques using nanoparticles shield antifungal agents, improving their stability and bioavailability, while minimizing drug degradation and systemic toxicity. Furthermore, patents often explore nanocomposite materials, blending nanoparticles with polymers or other substances to create multifunctional systems. These formulations aim to address challenges in drug resistance, biofilm formation, and the limited penetration of conventional antifungal therapies, offering promising solutions for various applications within the medical, agricultural, and industrial sectors.

**Table 6:** Patent of nanoparticle based antifungal drug delivery agents

S. no.	Patent no.	Patent title	Objective of invention	References
1	US20200155681A1	Delivery of nanoparticles	Delivery of formulations through photoactive plasmonic nanoparticles to treat skin tissue	[110]
2	JP6336902B2	Conjugate-based antifungal and antibacterial prodrugs	The non-active ingredient and targeting or modifying agents that are chemically bonded to the active ingredient are the key features of medicinal formulations	[111]
3	CN112996854A	High purity PEG lipids and uses thereof	The current disclosure offers high purity PEG lipids together with LNPs made of them, as well as instructions for utilising the LNPs for delivering therapeutic drugs to a subject	[112]
4	JP2022501359A	PEG lipids and their use	Compounds (i.e., PEG lipids) used in drug delivery systems, cosmetic compositions, and pharmaceutical compositions—such as those employed in lipid nanoparticle (LNP) formulations—are partially disclosed	[113]
5	JP7152549B2	Delivery of drug nanoparticles and methods of their use	A topical formulation designed to improve pharmaceutical nanoparticle skin penetration	[114]
6	JP6001640B2	Nanoparticles comprising esterified products of poly (methyl vinyl ether-co-maleic anhydride) and uses thereof	Half-sell-derived poly (methyl vinyl ether-co-maleic anhydride) (PVM/MA) nanoparticles, which exhibit strong bioadhesive properties to mucous membranes and good long-term stability in aqueous conditions, are included in the present invention	[115]
7	US8927018B2	Immobilized metallic nanoparticles as unique materials for therapeutic and biosensor applications	Metal nanoparticle-based surface modification methods that can be applied to a variety of polymeric or metal substrates	[116]
8	JP6412918B2	Multimodal silica nanoparticles	Fluorescent silica-based nanoparticles and techniques utilising the nanoparticles to identify, diagnose, or treat illnesses including cancer	[117]
9	JP6220389B2	Lipid nanoparticle compositions for delivering antisense oligonucleotides	Nucleic acids and related substances can be delivered via lipid nanoparticles	[118]
10	JP2007507489A	Water-soluble nanoparticle-encapsulated composite	An amphiphilic polymer envelops and encapsulates the active ingredient in an amorphous form, providing a hydrophilic encapsulated complex with a nano-dispersion of stable and water-soluble nano-sized particles	[119]
11	JP7028836B2	Antifungal topical composition and method of treatment	Topical formulations (and processes for making compositions) including polymers that can generate nanoparticles and antifungal drugs to treat fungal infections	[120]
12	AU2017206077B2	Mesoporous silica nanoparticles with lipid bilayer coating for cargo delivery	A silica body with a surface that defines many pores that can hold molecules is defined as part of a nanocarrier	[121]

### Different types of NPs with antifungal activity

**Fig. 7:**
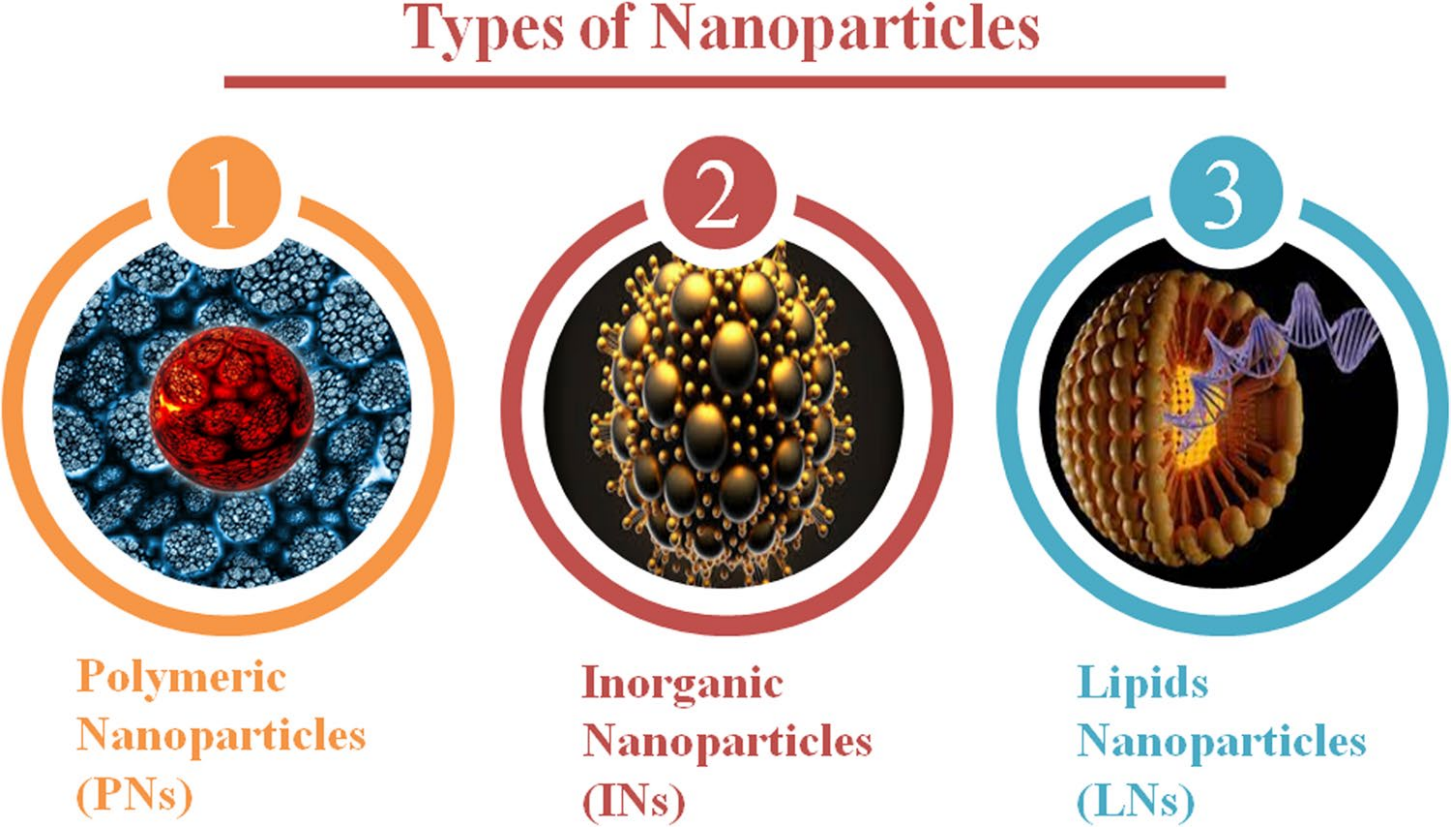
Types of nanoparticles

In a variety of fields of chemical science, electronics, photonics, supramolecular integration, delivery of medication, agriculture, and food company operations, nanotechnology has accomplished considerable achievements, more specifically, the application of "nanomedicine," or nanotechnology in medicine, has been a significant driving force behind the growth of several variations of drug-carrying NPs. NPs are classified into several classes based on their constituting material, sizes and shapes as represented in Fig. 7. Although the processes used to prepare NPs can produce a variety of NPs, each with a unique loading capacity, delivery, and shelf life [20]. Depending on how they appear, they are classified as dendrimers, nanospheres, nanocapsules, liposomes, micelles, polymerases, fullerenes, and nanotubes. They have been divided into groups that are organic and non-organic in other investigations. Within the organic category of NPs, the organic molecules serve as the primary building blocks, whereas in the mineral category, metals (such as iron, gold, etc.) and other mineral elements are crucial to the NPs structural integrity [122]. In contrast to mineral NPs, which have an organic coating over a mineral or metalcore, liposomes, dendrimers, carbon nanotubes, solid lipid NPs, and polymers are considered organic particles. The cores show electrical, magnetic, and fluorescent properties [123].

There is also another classification of NPs that divides NPs into two types of nanosphere and nanocapsules based on how they are created. These NPs are made of macromolecular or polymeric materials, either natural or synthetic. Nanocapsules are sac-like structures with a core chamber and a polymeric coating surrounding the medication. Nanospheres are a type of matrix system where drugs and polymers are either uniformly disseminated or surface-absorbed. When polymers are used as NPs, medications with specialized therapeutic properties for particular diseases including cancer are also present [10]. The use of nanomedicine in drug delivery and diagnostics is viewed as promising. Nanocarriers used as alternative medication delivery methods have attracted increased interest to date due to better drug penetration and distribution into targeted skin regions. Several investigations have examined the effective use of nanocarriers as diagnostic or therapeutic medications for life-threatening medical conditions.

#### Lipid nanoparticles (LNs)

**Fig. 8:**
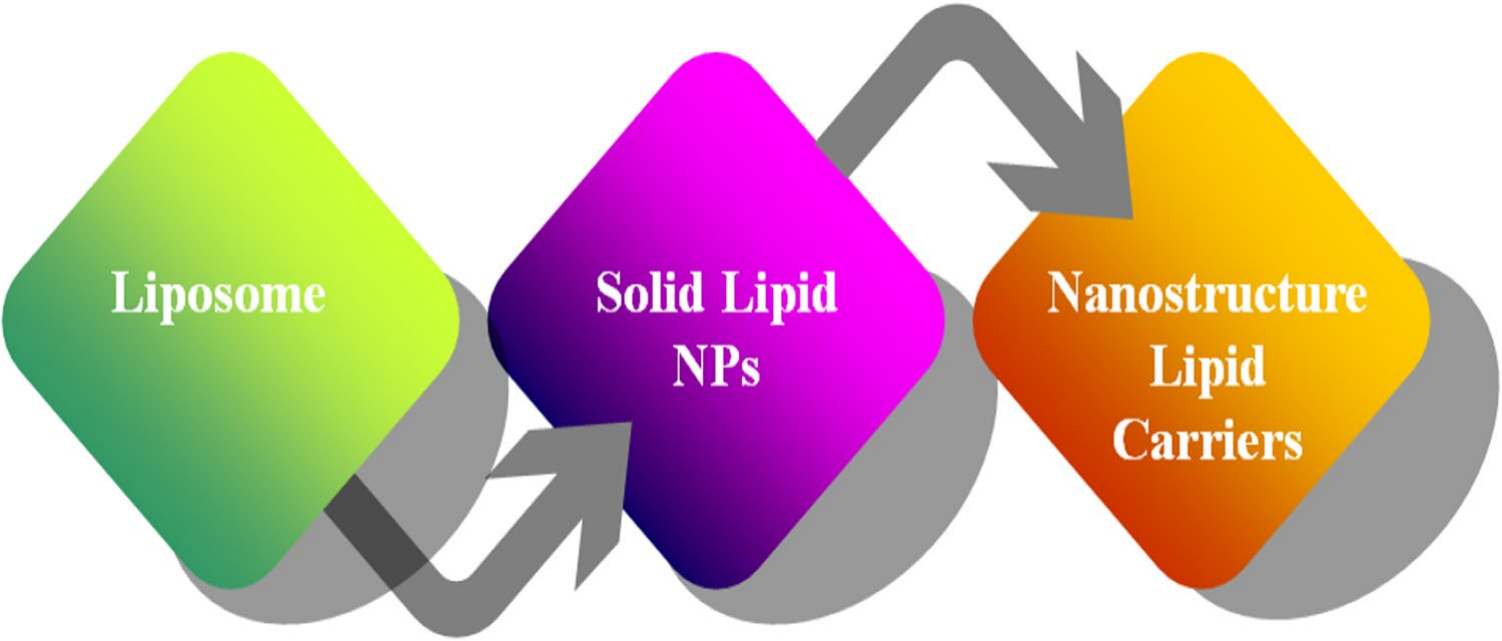
Types of lipid nanoparticles

LNs are good prospects as carriers for pharmaceuticals and food applications due to their distinctive physicochemical characteristics, which include taking the manner of presentation of solid core liposomes or other LNs that are highly biocompatible [124]. These LNs, which include solid cores or homogeneous lipid bilayers, can trap a variety of harmful substances. Lipid-based nanocarriers tends to be non-spherical due to the solvent's non-Polar nature of lipid hydrocarbon moieties or because of the solvent's electrostatic interaction with their polar/ionogenic phospholipid head [125]. Liposomes, solid lipid nanoparticles (SLNs), nanostructured lipid carriers (NLCs), and microemulsions are among the major types of lipid-based nanomaterials that are currently the focus of extensive research and clinical studies as mentioned in Fig. 8.

The second generation of lipid nanoparticles having a structure simulating a nanoemulsion is called NLC. SLN was the first generation of nanoparticles. NLC and SLN can be differentiate on the basis of their core [126]. They are both submicron-sized colloidal carriers that range in size from 40 to 1000 nm. Because NLC has the advantages in the drug loading and crystallinity issues, it is the most probable novel drug delivery technology compared to SLN. Solid and liquid lipids combine to generate NLC, so the quality of the product is improved over SLN. Three different kinds of structures exist in NLC: random, amorphous, and multiple. The irregular shape of the liquid lipid and its random structure including solid–liquid lipids improve the lipid layer's capacity to pass through the membrane. The amorphous structure is the second. It has a less crystalline character and can stop the loaded medicine from leaking. Multiple structures compose the third kind and have larger liquid lipid concentration than the other forms [127]. The majority of the excipients that are utilised in identified the NLC, which is biocompatible, non-irritating and biodegradable, are GRAS. NLC is a potentially effective drug delivery method that may be used for oral, pulmonary, ophthalmic, and central nervous system administration. The preparation and content of NLC might vary, which affects its stability parameters. The potential of NLC as a delivery method for different organs has been studied recently at the educational level [128].

##### Liposomes

Liposomes are the most commonly used LNs. Liposomes are the first phospholipid vesicle system which are invented in the 1960s, they possess a phospholipid bilayer homologous to the plasma membrane of human cells [129]. The liposomes can therefore facilitate medication has strong biocompatibility and plasma membrane drug diffusion [130]. Liposomes may be produced by a variety of techniques. Their dimensions, composition (varying phospholipids and cholesterol contents), charge (arising from the charges of the phospholipids composing them), and structure (either unilamellar liposomes, which comprise a single phospholipid bilayer encircling an aqueous compartment or multilamellar liposomes comprising multiple concentric bilayers separated by aqueous compartments) can all vary [131]. The properties of liposomes have a significant impact on their in vivo dispersion. It is possible to target specific cells or tissues using liposome-encapsulated medicines by covalently attaching monoclonal antibodies or other suitable proteins to the liposomes' outer surface. Phosphatidylethanolamine is a phospholipid molecule that may be included into phospholipid bilayers as one method of creating these immunoliposomes. Amino groups of the protein coupled with these molecules. When liposomes are administered to mammals, the majority of them naturally undergo phagocytosis. Long circulation half-lives may decrease or even prevent macrophage uptake and clearance when liposomes are used as drug depots (storage and slow release) or when liposomes are used to direct pharmaceuticals to nonphagocytic cells [132]. Liposomal self-assembly of an aqueous core has been enveloped by one or more circumferential lipid bilayers [133]. Liposomes commonly consist of a hydrophobic bilayer structure and an inner core that is hydrophilic and may vary in size from 20 nm up to more than 1 m. Because of their excellent capability to transport the both hydrophilic and hydrophobic medications in the lipid bilayer and aqueous lumen respectively, the versatility of liposomes is enhanced. Additionally, the liposome system offers the benefits of simple modification and the capacity for targeting, since it may be built with. The outermost layer of a cell, system, or tissue that has been changed with appropriate molecules (or ligands) to actively interact with the target molecule. However, it is challenging to produce substantial drug loading of hydrophobic medicines due to the constrained liposomes having a bilayer space. High drug loading must be balanced with liposome stability, particle size dispersion, and other factors. To enhance the clinical translation, additional investigation is require on targeted drug delivery utilizing nanocarriers to decrease toxic effects, increase the permeability and impacts of retention, and diminish the protein corona neuroprotective function [134]. Therefore, it is crucial to optimize the lipid bilayer composition, manufacturing processes, and physical characteristics.

##### Nano-structured lipid carriers (NLCs)

The delivery mechanisms for drugs based on lipids, referred to as NLCs, have been the subject of substantial studies. Because of their improved physical stability, drug-carrying capability, and bioavailability, they are believed to be superior to various other traditional lipid-based nanocarriers. At room temperature, they are solid in structure. A controlled combination of solid and liquid lipids with improved biological activity preservation and controlled release capabilities made comprised the lipid matrix of the NLCs. NLCs are biocompatible systems that stand out from other lipid-based formulations by having a stiff shape that gives them special features [135]. A number of lipids, comprising fatty acids, waxes, steroids, partial glycerides, and triglycerides, have been found in topical lipid nanoparticulate compositions. NLC does have a downside with the fact that the functionalization of the surface is challenging.

##### Solid lipid nanoparticles (SLNs)

SLNs have been invented in the 1990 to combine the advantageous characteristics of polymer nanocarriers, such as high drug loading capacity, controlled drug delivery, superior lipid emulsion biocompatibility, and enhanced drug bioavailability [136, 137]. SLNs can be produced using many different kinds of techniques, such as heat or cold the homogenization process which has good preparation reproducibility, is easy to scale up production, and doesn't use potentially harmful organic solvents [138]. The primary characteristic of SLNs is the presence of solid-at-room-temperature lipids. The preparation of SLNs systems frequently involves the use of biocompatible materials such as triglycerides, fatty acids, steroids, and bio waxes. The vast surface area and tiny size of SLNs make them ideal for covering with functionalized ligands, antibodies, and other functional groups. The potential use of SLNs in oral medication delivery systems is at the forefront among the various types of nanocarriers [139]. The ease of manufacture, pharmaceutical stability, higher drug content, efficient drug release, and high long-term stability are only a few benefits of SLNs. Additionally, because of its high lipid content, the SLNs system can effectively constitute anticancer medications and other compounds with low water solubility for drug administration. The potential use of SLNs in oral medication delivery systems is at the forefront among the various types of nanocarriers. The ease of manufacture, pharmaceutical stability, higher drug content, efficient drug release, and high long-term stability are only a few benefits of SLNs. Additionally, because of its high lipid content, the SLNs system can effectively constitute medications and other compounds with low aqueous solubility for drug administration. Phospholipids play a significant role in lipid and lipid-based drug delivery systems due to their many features, including amphiphilia, biocompatibility, and multifunctionality. But because of numerous drawbacks of liposomes, lipospheres, and microsimulation carrier system, including their challenging large-scale production, low percentage entrapment efficiency (% EE), and complex manufacturing processes, SLN delivery system has emerged. SLNs typically have a diameter of 50–1000 nm and a spherical shape. Lipids, which are solid at room temperature, emulsifiers—or occasionally a combination of the two—active pharmaceutical ingredients (APIs), and a suitable solvent system are the main components of SLN formulations. Various elements of nanocarrier-based drug delivery systems may be divided into subcategories based on factors including the administration route and degree of degradability [140]. The modes of administration are protein peptide delivery via nanoparticles as well as parenteral, oral, ocular, and topical administration of nanoparticles.

**Advantages of SLNs**

1. Avoiding organic solvents was possible with solid nanoparticles, which was first necessary with liposomes and polymeric nanoparticles.
2. In comparison to polymeric and liposomes, SLNs have superior reproducibility and large-scale manufacturing using a reasonable high pressure homogenization process.
3. The product's stability is enhanced for approximately 3 years when compared to polymeric nanoparticles, and it is more stable for the active ingredient than liposomes due to the rigid core lipid matrix.

#### Polymeric nanoparticles (PNs)

The 1970s brought the development of polymer NPs, which are more stable than liposomes and can be developed in similar ways. Additionally, polymer nanoparticle-controlled release characteristics are simpler to manage, and surface modification is practical [141]. Nanospheres and nano microcapsules are the two main kinds of polymer NPs. Despite nanospheres, which are "matrix type," in which drugs are distributed throughout the matrix, microcapsules are "repository type," in which medication drugs are present in a core encompassing by a shell of polymer. Due to their excellent biocompatibility, biodegradable nanoparticles have recently demonstrated tremendous potential as medication carriers as shown in Table 7. Although polymer NPs have a low rate of encapsulation, their large molecular weight makes them capable of quickly inducing an immunological response. Lipid polymer hybrid nanoparticles (LPNs) are a new carrier system that has been developed to address these drawbacks. It is obvious that the polymer regulates medication release and the lipid improves loading effectiveness and penetration. Consequently, LPNs have a significant chance of improving physical stability and biocompatibility. Several thrombolytic medicines are delivered orally for which the LPNs system has been investigated. In earlier investigations, chitosan and lipid nanoparticles were found to increase the oral bioavailability of heparin [142]. According to Khan, cisplatin-loaded lipid-polymer hybrid NPs are a promising platform for regulated cisplatin delivery in cancer therapy since they are an efficient delivery method for medication distribution at tumor locations [143].

**Table 7:** Shows the uses and production of polymeric Nanoparticles

S.N.	Polymeric nanoparticles	Uses	Production method	References
01	Nanocapsules PCL-NCs loaded with Amp B	Antifungal, anti-leishmanial	Nanoprecipitation method	[144]
02	Nanocapsules curcumin loaded PLGA NCs	Antifungal, antibacterial, pancreatic cancer	Nanoprecipitation method	[145]
03	Nanocapsule palmarosa oil loaded PCL-NCs	Antioxidant, antimicrobial	Nanoprecipitation method	[146]
04	Nanocapsule orange oil-loaded eudragit EPO NCs	Antimicrobial	Nanoprecipitation method	[147]
05	Nanocapsules ciprofloxacin loaded PLGA NCs	Antifungal/anti-inflammatory activity	Nanoprecipitation method	[148]
06	Nanosphere C-6 loaded polymeric cone shape NPs	Drug delivery	Emulsification	[149]
07	Nanosphere hyperfonin loaded AcDex-based NPs	Anti inflammatory activity	Emulsification	[150]

PCL, poly(ε-caprolactone) nanoparticles; NCs, nanocapsules; PLGA, poly(lactide-co-glycolide); AcDex, acetalated dextran

#### Inorganic nanoparticles (INs)

Inorganic NPs are non-toxic, hydrophilic, biocompatible, and extraordinarily stable when compared to organic compounds. To improve treatment effectiveness and reduce side effects, new materials, and therapy delivery methods have evolved together [151]. Thanks to developments in nanotechnology, other NPs of inorganic nature than calcium phosphates are currently introduced and are composed of effectual drug delivery matrices. Because inorganic NPs currently have incredibly improved chemical properties, they are frequently utilized as medication carriers [152].

There are several magnetic nanoparticles, gold nanoparticles, and semiconductor quantum dots the major inorganic NPs. There are many applications for inorganic NPs in medicine. The appealing attributes of NPs, such as their biocompatibility, adaptability, and special physical and chemical attributes, create them suitable for a range of applications in biomedicine. To move freely through the body, the created nanoparticle needs to be inactive, stable under physiological conditions, and have a surface that can be easily conjugated to the metal. The lung might be targeted with natural and inorganic NPs to lessen the negative impacts of conventional organization's elevated bloodstream groupings. By interfering with at least a single phase of the viral life cycle, carbon-based nanoparticles, oxides of metals (Fe2O3, TiO2, ZnO2), and transitions nanoparticles of metals (Ag, Cu, and Zn) demonstrate their intrinsic antipathogenic characteristics [153]**.** Inorganic nanoparticles include a wide range of compounds, such as fundamental metals (copper, gold, and silver), oxides of metal (titanium dioxide, copper oxide, zinc oxide, and iron oxide), metal salts, transition-metal dichalcogenides (molybdenum diselenide, and molybdenum disulphide), and inorganic non-metallic particle systems (tellurium, selenium, and carbon system) [154]. Due to their catalytic activity, nanoparticles of gold (AuNPs) are under investigation for a variety of prospective uses, whereas AgNPs, or silver nanoparticles, are used as a bactericide in a diversity of applications [155]. Both silver and gold nanoparticles feature the so-called surface plasmon, which gives them extraordinary aesthetic properties and considerably enhances the electric field on their surface, as a result of the electrons collective oscillation [156]. Among the inorganic and non-metallic elements, several chalcogens have been evaluated for the production of antifungal nanoparticles. Transition-metal dichalcogenide nanoparticles, or TMDC NPs, have recently been discovered to exhibit strong antibacterial activity. Molybdenum disulfide (MoS_2_) nanosheets have reportedly demonstrated specific antifungal properties While studies on antifungal MoSe_2_/CS nanosheets seem promising, studies on antifungal TMDC NPs have only recently started, and more study is necessary to determine the antifungal efficacy and biosafety of these NPs [157]. In addition to the NPs included in this study, numerous more interesting NP types have also been investigated as possible antifungal antifungal drugs. The noble metals palladium and platinum, which are also used to make nanoparticles, have antifungal properties similar to those of gold and silver. Platinum (Pt) nanoparticles (NPs) with diameters ranging from 10 to 50 nm were created by Velmurugan et al. and demonstrated effective antifungal activity against both *Cladosporium fulvum* and *Colletotrichum acutatum* [158]. Palladium (Pd) nanoparticles (NPs) ranging in mean size from 200 to 550 nm were synthesised, and their antifungal activities against *Fusarium oxysporum* and *Colletotrichum gloeosporioides* were studied [159]. In recent years, some bimetallic and trimetallic NPs with antifungal properties have been identified [160, 161]. Research has been done on a few additional metal oxide nanoparticles for antifungal purposes. Additionally, it has been observed that CuO NPs exhibit antifungal efficacy against *Aspergillus niger*, *A. flavus*, *Candida albicans*, and *C. glabrata* [162]. Potential antifungal activity against Fusarium oxysporum has been reported for magnesium oxide (MgO) and aluminium oxide (Al_2_O_3_) nanoparticles [163, 164]. Antifungal drug conjugated nanoparticles were also developed in laboratories. SiO_2_ NPs have moderate antifungal activity, however employing them as carriers of antifungal medications has shown excellent outcomes because of high porosity, large specific surface areas, and tuneable pore diameters in the micro-to mesopore range [165]. The synergistic antifungal effects of these inorganic nanoparticles with antifungal drugs, such as the release of mycotoxic ions, damage to membranes, proteins, DNA, and other essential cellular components, ROS overproduction, and ATP depletion, have been widely recognised.

## Future aspects and conclusion

Research in the field of nanomedicine has grown dramatically in the modern period. These topical medicines' incorporation of the nanocarrier idea offers several benefits. Compared to customary topical treatments. The future of skin healthcare is the application of nanomaterials for the treatment of skin cancer, tissue replacement, and quicker recovery from skin illnesses. Nanomaterials have recently been shown to be substantially more effective in skin tissue engineering research [166]. Due to improved skin penetration and targeted regulated drug distribution, nanomedicine-based preparations provide great therapeutic effects. Due to the inadequate effectiveness of many different contemporary anti-infective medications, treating a variety of superficial infections such dermatitis, acne, eczema, impetigo, pustular acne, and dermatitis keeps occurring to be the most challenging region surrounding research. That level of intricacy has not yet been attained by nanomedicine. The term "nano" refers to one billionth of a meter, and scientists can create nanomaterials as small as several nanometres, but current nanotechnology is unable to create functional electronic robots small enough to be safely injected into the bloodstream. However, since the 1970s, when the idea of nanotechnology was originally proposed, it has become a part of many things that we use daily, including electronics, fabrics, food, water and air purification systems, cosmetics, and medications. Many medical researchers were keen to apply nanotechnology to diagnose and cure diseases in light of these triumphs in various domains [167].

We briefly touch on the types of NPs employed for this purpose and their mechanisms of action in this review as we investigate the use of NPs in the research and development of new approaches to treatment and immunisations against systemic mycoses. Then, with a focus on Candida species, Cryptococcus species, Paracoccidioides species, Histoplasma species, Coccidioides species, and Aspergillus species, we describe the state of the art of NPs for the development of novel antifungal drugs and vaccines aimed in opposition to systemic mycoses**.** Particle size distribution, release rates, targeting efficiencies, and toxicities all depend critically on the optimum particle size. Because of the relationship between particle size and skin permeability, content can be delivered to the epidermal and dermal layers more effectively by nanovesicles with a diameter of 70 nm or less than by those with a diameter of 300 nm [168]. Smaller NPs have the tendency to remain on the skin's surface rather than penetrating into the deeper layers. Skin diseases such aspergillosis, dermatophytosis, onychomycosis, and candidiasis may be treated with NPs. Particle size, skin condition, composition, and transcellular or intracellular pathways all have a significant impact on their penetration. Zeta potential, molecular weight, encapsulation frequency, and chitosan deacetylation degree all influence particle size. Additional research can be conducted to examine the impact of these characteristics on the effectiveness of transdermal medication administration. NPs have demonstrated a lot of promise as medication transporters, but it's crucial to carefully assess their efficacy and safety over the long run. Some other important facts such as enhanced targeted drug delivery, sustained release, improved penetration and reduced side effects make more effective as compared to conventional method [169]. Following studies concentrates on NPs based antifungal drug delivery, tissue targeting, and drug loading capacity which focus on the maximum drug bioavailability of ointment. Current developments of NPs in medical purpose include the use of additives of antifungal agents for determining, and treating the pathogens, and diseases described briefly. Nevertheless, much remains to be learned about NP-assisted antifungal drugs.

## Abbreviations

NPs: Nanoparticles
LNs: Lipid nanoparticles
NLCs: Nano-structured lipid carriers
SLNs: Solid lipid nanoparticles
INs: Inorganic nanoparticles
PNs: Polymeric nanoparticles
CNS: Central nervous system
MDR: Multi drug resistant
FLZ: Fluconazole
C.: Candida
SC: Stratum corneum

## References

[1] Tiew PY, Mac Aogain M, Ali NABM, Thng KX, Goh K, Lau KJX, Chotirmall SH (2020). The mycobiome in health and disease: emerging concepts, methodologies and challenges. Mycopathologia.

[2] Gnat S, Łagowski D, Nowakiewicz A, Dyląg M (2021). A global view on fungal infections in humans and animals: infections caused by dimorphic fungi and dermatophytoses. J Appl Microbiol.

[3] Gnat S, Łagowski D, Nowakiewicz A, Dyląg M, Osińska M, Sawicki M (2021). Detection and identification of dermatophytes based on currently available methods—a comparative study. J Appl Microbiol.

[4] Bever JD, Morton JB, Antonovics J, Schultz PA (1996). Host-dependent sporulation and species diversity of arbuscular mycorrhizal fungi in a mown grassland. J Ecol.

[5] Brunet K, Alanio A, Lortholary O, Rammaert B (2018). Reactivation of dormant/latent fungal infection. J Infect.

[6] Zmeili OS, Soubani AO (2007). Pulmonary aspergillosis: a clinical update. QJM.

[7] Lengert EV, Talnikova EE, Tuchin VV, Svenskaya YI (2020). Prospective nanotechnology-based strategies for enhanced intra- and transdermal delivery of antifungal drugs. Skin Pharmacol Physiol.

[8] Hart R, Bell-Syer SEM, Crawford F, Torgerson DJ, Young P, Russell I (1999). Systematic review of topical treatments for fungal infections of the skin and nails of the feet. BMJ.

[9] Gupta AK, Cooper EA (2008). Update in antifungal therapy of dermatophytosis. Mycopathologia.

[10] Nami S, Aghebati-Maleki A, Aghebati-Maleki L (2021). Current applications and prospects of nanoparticles for antifungal drug delivery. EXCLI J.

[11] Thiesen B, Jordan A (2008). Clinical applications of magnetic nanoparticles for hyperthermia. Int J Hyperth.

[12] Pankhurst QA, Connolly J, Jones SK, Dobson J (2003). Applications of magnetic nanoparticles in biomedicine. J Phys D Appl Phys.

[13] Langer R, Folkman J (1976). Polymers for the sustained release of proteins and other macromolecules. Nature.

[14] Salata O (2004). Application of nanoparticles in biology and medicine. J Nanobiotechnol.

[15] Gupta R, Xie H (2018). Nanoparticles in Daily Life: applications, toxicity and regulations. J Environ Pathol Toxicol Oncol.

[16] Mura S, Nicolas J, Couvreur P (2013). Stimuli-responsive nanocarriers for drug delivery. Nat Mater.

[17] Pantarotto D, Partidos CD, Hoebeke J, Brown F, Kramer E, Briand J-P, Muller S, Prato M, Bianco A (2003). Immunization with peptide-functionalized carbon nanotubes enhances virus-specific neutralizing antibody responses. Chem Biol.

[18] Mahapatro A, Singh DK (2011). Biodegradable nanoparticles are excellent vehicle for site directed in-vivo delivery of drugs and vaccines. J Nanobiotechnol.

[19] Tassa C, Shaw SY, Weissleder R (2011). Dextran-coated iron oxide nanoparticles: a versatile platform for targeted molecular imaging, molecular diagnostics, and therapy. Acc Chem Res.

[20] Hu C-MJ, Aryal S, Zhang L (2010). Nanoparticle-assisted combination therapies for effective cancer treatment. Ther Deliv.

[21] Abpeikar Z, Safaei M, Akbar Alizadeh A, Goodarzi A, Hatam G (2023). The novel treatments based on tissue engineering, cell therapy and nanotechnology for cutaneous leishmaniasis. Int J Pharm.

[22] Stoimenov PK, Klinger RL, Marchin GL, Klabunde KJ (2002). Metal oxide nanoparticles as bactericidal agents. Langmuir.

[23] Yamamoto O (2001). Influence of particle size on the antibacterial activity of zinc oxide. Int J Inorg Mater.

[24] Sonia S, Linda Jeeva Kumari H, Ruckmani K, Sivakumar M (2017). Antimicrobial and antioxidant potentials of biosynthesized colloidal zinc oxide nanoparticles for a fortified cold ointment formulation: a potent nanocosmeceutical application. Mater Sci Eng C.

[25] Catalano A, Iacopetta D, Ceramella J, Scumaci D, Giuzio F, Saturnino C, Aquaro S, Rosano C, Sinicropi MS (2022). Multidrug resistance (MDR): a widespread phenomenon in pharmacological therapies. Molecules.

[26] Perni S, Prokopovich P, Pratten J, Parkin IP, Wilson M (2011). Nanoparticles: their potential use in antibacterial photodynamic therapy. Photochem Photobiol Sci.

[27] Rai M, Ingle AP, Gaikwad S, Gupta I, Gade A, Silvério Da Silva S (2016). Nanotechnology based anti-infectives to fight microbial intrusions. J Appl Microbiol.

[28] Kumar PPNV, Pammi SVN, Kollu P, Satyanarayana KVV, Shameem U (2014). Green synthesis and characterization of silver nanoparticles using Boerhaavia diffusa plant extract and their anti bacterial activity. Ind Crops Prod.

[29] Chernousova S, Epple M (2013). Silver as antibacterial agent: ion, nanoparticle, and metal. Angew Chem Int Ed.

[30] Adebayo-Tayo BC, Borode SO, Alao SO (2022). In-vitro antibacterial and antifungal efficacy of greenly fabricated Senna alata leaf extract silver nanoparticles and silver nanoparticle-ointment blend. Period Polytech Chem Eng.

[31] Begum J, Mir NA, Lingaraju MC, Buyamayum B, Dev K (2020). Recent advances in the diagnosis of dermatophytosis. J Basic Microbiol.

[32] Goldstein AO, Smith KM, Ives TJ, Goldstein B. Mycotic infections. Effective management of conditions involving the skin, hair, and nails. Geriatrics. 2000;55(5):40–2, 45–7, 51-2. 10826264

[33] Kane J, Krajden S, Summerbell RC, Sibbald RG (1990). Infections caused by *Trichophyton raubitschekii*: clinical and epidemiological features. Mycoses.

[34] Chakrabarti A, Bonifaz A, Gutierrez-Galhardo MC, Mochizuki T, Li S (2015). Global epidemiology of sporotrichosis. Med Mycol.

[35] Bienvenu A-L, Picot S (2020). Mycetoma and chromoblastomycosis: perspective for diagnosis improvement using biomarkers. Molecules.

[36] Queiroz-Telles F, McGinnis MR, Salkin I, Graybill JR (2003). Subcutaneous mycoses. Infect Dis Clin N Am.

[37] Kyle AA, Dahl MV (2004). Topical therapy for fungal infections. Am J Clin Dermatol.

[38] Garg A, Sharma GS, Goyal AK, Ghosh G, Si SC, Rath G (2020). Recent advances in topical carriers of anti-fungal agents. Heliyon.

[39] Gupta M, Agrawal U, Vyas SP (2012). Nanocarrier-based topical drug delivery for the treatment of skin diseases. Expert Opin Drug Deliv.

[40] Ząbek A, Nagaj J, Grabowiecka A, Dworniczek E, Nawrot U, Młynarz P, Jeżowska-Bojczuk M (2015). Activity of fluconazole and its Cu(II) complex towards Candida species. Med Chem Res.

[41] Khalid A, Ahmed N, Qindeel M, Asad MI, Khan GM, ur.Rehman A (2021). Development of novel biopolymer-based nanoparticles loaded ointment for potential treatment of topical fungal infections. Drug Dev Ind Pharm.

[42] Dornburg A, Townsend JP, Wang Z, Townsend J, Wang Z (2017). Maximizing power in phylogenetics and phylogenomics: a perspective illuminated by fungal big data. Advances in genetics.

[43] Siyal AL. Cell its structure and functions; 2019. 10.13140/RG.2.2.18777.06244.

[44] Blackwell M (2011). The fungi: 1, 2, 3 … 5.1 million species?. Am J Bot.

[45] Bard J, Burger A, Davidson D, Baldock R (2008). Anatomical ontologies for model organisms: the fungi and animals. Anatomy ontologies for bioinformatics.

[46] Werner GDA, Cornwell WK, Sprent JI, Kattge J, Kiers ET (2014). A single evolutionary innovation drives the deep evolution of symbiotic N2-fixation in angiosperms. Nat Commun.

[47] Lee PP, Lau Y-L (2017). Cellular and molecular defects underlying invasive fungal infections—revelations from endemic mycoses. Front Immunol.

[48] Perlroth J, Choi B, Spellberg B (2007). Nosocomial fungal infections: epidemiology, diagnosis, and treatment. Med Mycol.

[49] Hay RJ (2006). Fungal infections. Clin Dermatol.

[50] Reddy GKK, Padmavathi AR, Nancharaiah YV (2022). Fungal infections: pathogenesis, antifungals and alternate treatment approaches. Curr Res Microb Sci.

[51] Ameen M (2010). Epidemiology of superficial fungal infections. Clin Dermatol.

[52] La Hoz RM, Baddley JW (2012). Subcutaneous fungal infections. Curr Infect Dis Rep.

[53] Hay RJ, Williams HC, Bigby M, Herxheimer A, Naldi L, Rzany B, Dellavalle RP, Ran Y, Furue M (2014). Deep fungal infections. Evidence-based dermatology.

[54] Houšť J, Spížek J, Havlíček V (2020). Antifungal drugs. Metabolites.

[55] Hokken MWJ, Zwaan BJ, Melchers WJG, Verweij PE (2019). Facilitators of adaptation and antifungal resistance mechanisms in clinically relevant fungi. Fungal Genet Biol.

[56] Imam SS, Gilani SJ, Zafar A, Jumah MNB, Alshehri S (2023). Formulation of miconazole-loaded chitosan–carbopol vesicular gel: optimization to in vitro characterization, irritation, and antifungal assessment. Pharmaceutics.

[57] Rençber S, Karavana SY, Yilmaz FF, Eraç B, Nenni M, Gurer-Orhan H, Limoncu MH, Güneri P, Ertan G (2019). Formulation and evaluation of fluconazole loaded oral strips for local treatment of oral candidiasis. J Drug Deliv Sci Technol.

[58] Bouchand C, Nguyen D, Secretan P-H, Vidal F, Guery R, Auvity S, Cohen JF, Lanternier F, Lortholary O, Cisternino S, Schlatter J (2020). Voriconazole topical cream formulation: evidence for stability and antifungal activity. Int J Antimicrob Agents.

[59] Shirsand S, Para M, Nagendrakumar D, Kanani K, Keerthy D (2012). Formulation and evaluation of Ketoconazole niosomal gel drug delivery system. Int J Pharm Investig.

[60] Khatter NJ, Khan MA. Clotrimazole. In: StatPearls. Treasure Island, FL: StatPearls Publishing; 2023.

[61] Srivastava S, Mahor A, Singh G, Bansal K, Singh PP, Gupta R, Dutt R, Alanazi AM, Khan AA, Kesharwani P (2021). Formulation development, in vitro and in vivo evaluation of topical hydrogel formulation of econazole nitrate-loaded β-cyclodextrin nanosponges. J Pharm Sci.

[62] Banerjee M, Ghosh A, Basak S (2012). Comparative evaluation of efficacy and safety of topical fluconazole and clotrimazole in the treatment of Tineacorporis. J Pak Assoc Dermatol.

[63] Borgers M (1980). Mechanism of action of antifungal drugs, with special reference to the imidazole derivatives. Clin Infect Dis.

[64] Soliman GM (2017). Nanoparticles as safe and effective delivery systems of antifungal agents: achievements and challenges. Int J Pharm.

[65] Priyanka P, Sri Rekha M, Devi AS (2022). Review on formulation and evaluation of solid lipid nanoparticles for vaginal application. Int J Pharm Pharm Sci.

[66] León-Buitimea A, Garza-Cervantes JA, Gallegos-Alvarado DY, Osorio-Concepción M, Morones-Ramírez JR (2021). Nanomaterial-based antifungal therapies to combat fungal diseases Aspersgillosis, Coccidioidomycosis, Mucormycosis, and Candidiasis. Pathogens.

[67] Gudikandula K, CharyaMaringanti S (2016). Synthesis of silver nanoparticles by chemical and biological methods and their antimicrobial properties. J Exp Nanosci.

[68] Lara HH, Ixtepan-Turrent L, Jose Yacaman M, Lopez-Ribot J (2020). Inhibition of *Candida auris* Biofilm formation on medical and environmental surfaces by silver nanoparticles. ACS Appl Mater Interfaces.

[69] Vazquez-Munoz R, Lopez FD, Lopez-Ribot JL (2020). Silver nanoantibiotics display strong antifungal activity against the emergent multidrug-resistant yeast candida auris under both planktonic and biofilm growing conditions. Front Microbiol.

[70] Arshad HM, Shahzad A, Shahid S, Ali S, Rauf A, Sharif S, Ullah ME, Ullah MI, Ali M, Ahmad HI (2022). Synthesis and biomedical applications of zirconium nanoparticles: advanced leaps and bounds in the recent past. Biomed Res Int.

[71] Zhang ML, Feng C, Zhang WX, Luan XW, Jiang J, Li LF (2013). Synthesis of bismuth nanoparticles by a simple one-step solvothermal reduction route. Appl Mech Mater.

[72] Vazquez-Munoz R, Lopez FD, Lopez-Ribot JL (2020). Bismuth nanoantibiotics display anticandidal activity and disrupt the biofilm and cell morphology of the emergent pathogenic yeast Candida auris. Antibiotics.

[73] Asamoah RB, Yaya A, Mensah B, Nbalayim P, Apalangya V, Bensah YD, Damoah LNW, Agyei-Tuffour B, Dodoo-Arhin D, Annan E (2020). Synthesis and characterization of zinc and copper oxide nanoparticles and their antibacteria activity. Results Mater.

[74] Mohamed AA, Abu-Elghait M, Ahmed NE, Salem SS (2021). Eco-friendly mycogenic synthesis of ZnO and CuO nanoparticles for in vitro antibacterial, antibiofilm, and antifungal applications. Biol Trace Elem Res.

[75] Hammami I, Alabdallah NM, Jomaa AA, Kamoun M (2021). Gold nanoparticles: synthesis properties and applications. J King Saud Univ Sci.

[76] El-Kemary M, Nagy N, El-Mehasseb I (2013). Nickel oxide nanoparticles: synthesis and spectral studies of interactions with glucose. Mater Sci Semicond Process.

[77] Nasrollahzadeh M, Sajjadi M, Iravani S, Varma RS (2020). Trimetallic nanoparticles: greener synthesis and their applications. Nanomaterials.

[78] Cleare LG, Li KL, Abuzeid WM, Nacharaju P, Friedman JM, Nosanchuk JD (2020). NO Candida auris: nitric oxide in nanotherapeutics to combat emerging fungal pathogen Candida auris. J Fungi.

[79] Cao Z, Spilker T, Fan Y, Kalikin LM, Ciotti S, LiPuma JJ, Makidon PE, Wilkinson JE, Baker JR, Wang SH (2017). Nanoemulsion is an effective antimicrobial for methicillin-resistant *Staphylococcus aureus* in infected wounds. Nanomedicine.

[80] Mustafa IF, Hussein MZ (2020). Synthesis and technology of nanoemulsion-based pesticide formulation. Nanomaterials.

[81] Mohamed DY (2018). Detection the antifungal effect of zirconium oxide nanoparticles on mold which isolated from domestic’s bathroom. Al-Mustansiriyah J Sci.

[82] Betancourt-Galindo R, Reyes-Rodriguez PY, Puente-Urbina BA, Avila-Orta CA, Rodríguez-Fernández OS, Cadenas-Pliego G, Lira-Saldivar RH, García-Cerda LA (2014). Synthesis of copper nanoparticles by thermal decomposition and their antimicrobial properties. J Nanomater.

[83] Garse H, Jagtap P, Kadam V (2015). Solid lipid nanoparticles based gel for topical delivery of antifungal agent. Int J Pharm Sci Res.

[84] Sojinrin T, Conde J, Liu K, Curtin J, Byrne HJ, Cui D, Tian F (2017). Plasmonic gold nanoparticles for detection of fungi and human cutaneous fungal infections. Anal Bioanal Chem.

[85] Aldosari MA, Darwish SS, Adam MA, Elmarzugi NA, Ahmed SM (2019). Using ZnO nanoparticles in fungal inhibition and self-protection of exposed marble columns in historic sites. Archaeol Anthropol Sci.

[86] Hasheminejad N, Khodaiyan F, Safari M (2019). Improving the antifungal activity of clove essential oil encapsulated by chitosan nanoparticles. Food Chem.

[87] Humisto A, Jokela J, Teigen K, Wahlsten M, Permi P, Sivonen K, Herfindal L (2019). Characterization of the interaction of the antifungal and cytotoxic cyclic glycolipopeptide hassallidin with sterol-containing lipid membranes. Biochim Biophys Acta (BBA) Biomembr.

[88] Alhowyan AA, Altamimi MA, Kalam MA, Khan AA, Badran M, Binkhathlan Z, Alkholief M, Alshamsan A (2019). Antifungal efficacy of Itraconazole loaded PLGA-nanoparticles stabilized by vitamin-E TPGS: in vitro and ex vivo studies. J Microbiol Methods.

[89] Spadari CDC, De Bastiani FWMDS, Lopes LB, Ishida K (2019). Alginate nanoparticles as non-toxic delivery system for miltefosine in the treatment of candidiasis and cryptococcosis. Int J Nanomed.

[90] Fernandes Costa A, Evangelista Araujo D, Santos Cabral M, TelesBrito I, Borges De MenezesLeite L, Pereira M, Correa Amaral A (2019). Development, characterization, and *in vitro–in vivo* evaluation of polymeric nanoparticles containing miconazole and farnesol for treatment of vulvovaginal candidiasis. Med Mycol.

[91] Roque L, Castro P, Molpeceres J, Viana AS, Roberto A, Reis C, Rijo P, Tho I, Sarmento B, Reis C (2018). Bioadhesive polymeric nanoparticles as strategy to improve the treatment of yeast infections in oral cavity: In-vitro and ex-vivo studies. Eur Polym J.

[92] Yang M, Xie S, Adhikari VP, Dong Y, Du Y, Li D (2018). The synergistic fungicidal effect of low-frequency and low-intensity ultrasound with amphotericin B-loaded nanoparticles on C. albicans in vitro. Int J Pharm.

[93] Zambom CR, Da Fonseca FH, Crusca E, Da Silva PB, Pavan FR, Chorilli M, Garrido SS (2019). A novel antifungal system with potential for prolonged delivery of histatin 5 to limit growth of Candida albicans. Front Microbiol.

[94] Khan WA, Sharma V, Maurya P, Bijauliya RK (2019). Development and characterization of oxiconazole nitrate loaded ethosomal gel for treating fungal infection. World J Pharmacol Res.

[95] Fetih G (2016). Fluconazole-loaded niosomal gels as a topical ocular drug delivery system for corneal fungal infections. J Drug Deliv Sci Technol.

[96] Guo F, Wang J, Ma M, Tan F, Li N (2015). Skin targeted lipid vesicles as novel nano-carrier of ketoconazole: characterization, in vitro and in vivo evaluation. J Mater Sci Mater Med.

[97] Laurent A, Pantet O, Laurent L, Hirt-Burri N, De Buys Roessingh A, Raffoul W, Laurent P, Monod M, Applegate LA (2020). Potency and stability of liposomal Amphotericin B formulated for topical management of Aspergillus spp. infections in burn patients. Burns Open.

[98] Nafisi S, Maibach HI. Skin penetration of nanoparticles. In: Emerging nanotechnologies in immunology, pp 47–88. Elsevier; 2018. 10.1016/B978-0-323-40016-9.00003-8

[99] Hu Y, Zhang W, Chu X, Wang A, He Z, Si C-L, Hu W (2023). Dendritic cell-targeting polymer nanoparticle-based immunotherapy for cancer: a review. Int J Pharm.

[100] Desai P, Patlolla RR, Singh M (2010). Interaction of nanoparticles and cell-penetrating peptides with skin for transdermal drug delivery. Mol Membr Biol.

[101] Ataide JA, Coco JC, Dos Santos ÉM, Beraldo-Araujo V, Silva JRA, De Castro KC, Lopes AM, Filipczak N, Yalamarty SSK, Torchilin VP, Mazzola PG (2023). Co-encapsulation of drugs for topical application—a review. Molecules.

[102] Ajaz S, Ahmed T, Shahid M, Noman M, Shah AA, Mehmood MA, Abbas A, Cheema AI, Iqbal MZ, Li B (2021). Bioinspired green synthesis of silver nanoparticles by using a native Bacillus sp. strain AW1–2: characterization and antifungal activity against Colletotrichum falcatum Went. Enzyme Microb Technol.

[103] Khan T, Yasmin A, Townley HE (2020). An evaluation of the activity of biologically synthesized silver nanoparticles against bacteria, fungi and mammalian cell lines. Colloids Surf B Biointerfaces.

[104] Vazquez-Muñoz R, Avalos-Borja M, Castro-Longoria E (2014). Ultrastructural analysis of candida albicans when exposed to silver nanoparticles. PLoS ONE.

[105] Wang L, He D, Gao S, Wang D, Xue B, Yokoyama K (2016). Biosynthesis of silver nanoparticles by the fungus Arthroderma fulvum and its antifungal activity against genera of Candida, Aspergillus and Fusarium. IJN.

[106] Mussin J, Robles-Botero V, Casañas-Pimentel R, Rojas F, Angiolella L, San Martín-Martínez E, Giusiano G (2021). Antimicrobial and cytotoxic activity of green synthesis silver nanoparticles targeting skin and soft tissue infectious agents. Sci Rep.

[107] Mussin J, Giusiano G (2022). Biogenic silver nanoparticles as antifungal agents. Front Chem.

[108] Soares MRPS, Corrêa RO, Stroppa PHF, Marques FC, Andrade GFS, Corrêa CC, Brandão MAF, Raposo NRB (2018). Biosynthesis of silver nanoparticles using Caesalpinia ferrea (Tul.) Martius extract: physicochemical characterization, antifungal activity and cytotoxicity. PeerJ.

[109] Salati S, Doudi M, Madani M (2018). The biological synthesis of silver nanoparticles by mango plant extract and its anti-Candida effects. J Appl Biotechnol Rep.

[110] Patent No. US20200155681A1.

[111] Patent No. JP6336902B2.

[112] Patent No. CN112996854A.

[113] Patent No. JP2022501359A.

[114] Patent No. JP7152549B2.

[115] Patent No. JP6001640B2.

[116] Patent No. US8927018B2.

[117] Patent No. JP6412918B2.

[118] Patent No. JP6220389B2.

[119] Patent No. JP2007507489A.

[120] Patent No. JP7028836B2.

[121] Patent No. AU2017206077B2.

[122] Erdoğar N, Akkın S, Bilensoy E (2019). Nanocapsules for drug delivery: an updated review of the last decade. Recent Pat Drug Deliv Formul.

[123] Frank LA, Contri RV, Beck RCR, Pohlmann AR, Guterres SS (2015). Improving drug biological effects by encapsulation into polymeric nanocapsules: improving drug effects by nanocapsules. Wiley Interdiscip Rev Nanomed Nanobiotechnol.

[124] Wacker M (2013). Nanocarriers for intravenous injection—the long hard road to the market. Int J Pharm.

[125] Jahangir MA, Taleuzzaman M, Kala C, Gilani SJ (2020). Advancements in polymer and lipid-based nanotherapeutics for cancer drug targeting. Curr Pharm Des.

[126] Ranpise NS, Korabu SS, Ghodake VN (2014). Second generation lipid nanoparticles (NLC) as an oral drug carrier for delivery of lercanidipine hydrochloride. Colloids Surf B Biointerfaces.

[127] Khan S, Sharma A, Jain V (2023). An overview of nanostructured lipid carriers and its application in drug delivery through different routes. Adv Pharm Bull.

[128] Kumar Sahoo P, Neha SL, Pannu A (2020). An overview of second generation nanoparticles—nanostructure lipid carrier drug delivery system. PCI-Approved-IJPSN.

[129] Allen TM, Cullis PR (2013). Liposomal drug delivery systems: from concept to clinical applications. Adv Drug Deliv Rev.

[130] Guimarães D, Cavaco-Paulo A, Nogueira E (2021). Design of liposomes as drug delivery system for therapeutic applications. Int J Pharm.

[131] Nsairat H, Khater D, Sayed U, Odeh F, Al Bawab A, Alshaer W (2022). Liposomes: structure, composition, types, and clinical applications. Heliyon.

[132] Derakhshandeh K, Kashanian, Hemati Azandaryani A (2011). New surface-modified solid lipid nanoparticles using N-glutaryl phosphatidylethanolamine as the outer shell. IJN.

[133] Raemdonck K, Braeckmans K, Demeester J, De Smedt SC (2014). Merging the best of both worlds: hybrid lipid-enveloped matrix nanocomposites in drug delivery. Chem Soc Rev.

[134] Cheng Z, Li M, Dey R, Chen Y (2021). Nanomaterials for cancer therapy: current progress and perspectives. J Hematol Oncol.

[135] Muller H, R., Shegokar, R., & M. Keck, C. (2011). 20 Years of lipid nanoparticles (SLN & NLC): present state of development & industrial applications. Curr Drug Discov Technol.

[136] Muller R (2000). Solid lipid nanoparticles (SLN) for controlled drug delivery—a review of the state of the art. Eur J Pharm Biopharm.

[137] Yuan H, Wang L-L, Du Y-Z, You J, Hu F-Q, Zeng S (2007). Preparation and characteristics of nanostructured lipid carriers for control-releasing progesterone by melt-emulsification. Colloids Surf B.

[138] Sivadasan D, Ramakrishnan K, Mahendran J, Ranganathan H, Karuppaiah A, Rahman H (2023). Solid lipid nanoparticles: applications and prospects in cancer treatment. Int J Mol Sci.

[139] Naseri N, Valizadeh H, Zakeri-Milani P (2015). Solid lipid nanoparticles and nanostructured lipid carriers: structure, preparation and application. Adv Pharm Bull.

[140] Duan Y, Dhar A, Patel C, Khimani M, Neogi S, Sharma P, Siva Kumar N, Vekariya RL (2020). A brief review on solid lipid nanoparticles: part and parcel of contemporary drug delivery systems. RSC Adv.

[141] Patel T, Zhou J, Piepmeier JM, Saltzman WM (2012). Polymeric nanoparticles for drug delivery to the central nervous system. Adv Drug Deliv Rev.

[142] Hallan SS, Nidhi, Kaur V, Jain V, Mishra N (2017). Development and characterization of polymer lipid hybrid nanoparticles for oral delivery of LMWH. Artif Cells Nanomed Biotechnol.

[143] Khan MM, Madni A, Torchilin V, Filipczak N, Pan J, Tahir N, Shah H (2019). Lipid-chitosan hybrid nanoparticles for controlled delivery of cisplatin. Drug Deliv.

[144] Saqib M, Ali Bhatti AS, Ahmad NM, Ahmed N, Shahnaz G, Lebaz N, Elaissari A (2020). Amphotericin B loaded polymeric nanoparticles for treatment of leishmania infections. Nanomaterials (Basel).

[145] Zakaria H, Kurdi RE, Patra D (2022). Curcumin-PLGA based nanocapsule for the fluorescence spectroscopic detection of dopamine. RSC Adv.

[146] Jummes B, Sganzerla WG, da Rosa CG, Noronha CM, Nunes MR, Bertoldi FC, Barreto PLM (2020). Antioxidant and antimicrobial poly-ε-caprolactone nanoparticles loaded with Cymbopogon martinii essential oil. Biocatal Agric Biotechnol.

[147] Froiio F, Ginot L, Paolino D, Lebaz N, Bentaher A, Fessi H, Elaissari A (2019). Essential oils-loaded polymer particles: preparation, characterization and antimicrobial property. Polymers (Basel).

[148] Günday Türeli N, Torge A, Juntke J, Schwarz BC, Schneider-Daum N, Türeli AE, Lehr C-M, Schneider M (2017). Ciprofloxacin-loaded PLGA nanoparticles against cystic fibrosis P. aeruginosa lung infections. Eur J Pharm Biopharm.

[149] Zielińska A, Carreiró F, Oliveira AM, Neves A, Pires B, Venkatesh DN, Durazzo A, Lucarini M, Eder P, Silva AM, Santini A, Souto EB (2020). Polymeric nanoparticles: production, characterization, toxicology and ecotoxicology. Molecules.

[150] Traeger A, Voelker S, Shkodra-Pula B, Kretzer C, Schubert S, Gottschaldt M, Schubert US, Werz O (2020). Improved bioactivity of the natural product 5-lipoxygenase inhibitor hyperforin by encapsulation into polymeric nanoparticles. Mol Pharm.

[151] Choi S-J, Lee JK, Jeong J, Choy J-H (2013). Toxicity evaluation of inorganic nanoparticles: considerations and challenges. Mol Cell Toxicol.

[152] Kong F-Y, Zhang J-W, Li R-F, Wang Z-X, Wang W-J, Wang W (2017). Unique roles of gold nanoparticles in drug delivery, targeting and imaging applications. Molecules.

[153] Prusty K, Swain SK (2018). Nano silver decorated polyacrylamide/dextran nanohydrogels hybrid composites for drug delivery applications. Mater Sci Eng C.

[154] Huang T, Li X, Maier M, O’Brien-Simpson NM, Heath DE, O’Connor AJ (2023). Using inorganic nanoparticles to fight fungal infections in the antimicrobial resistant era. Acta Biomater.

[155] Marcu A, Pop S, Dumitrache F, Mocanu M, Niculite CM, Gherghiceanu M, Lungu CP, Fleaca C, Ianchis R, Barbut A, Grigoriu C, Morjan I (2013). Magnetic iron oxide nanoparticles as drug delivery system in breast cancer. Appl Surf Sci.

[156] Koul B, Poonia AK, Yadav D, Jin J-O (2021). Microbe-mediated biosynthesis of nanoparticles: applications and future prospects. Biomolecules.

[157] Basu P, Chakraborty J, Ganguli N, Mukherjee K, Acharya K, Satpati B, Khamrui S, Mandal S, Banerjee D, Goswami D, Nambissan PMG, Chatterjee K. Defect-engineered MoS_2_ nanostructures for reactive oxygen species generation in the dark: antipollutant and antifungal performances. ACS Appl Mater Interfaces. 2019;11(51):48179–91. 10.1021/acsami.9b12988. 10.1021/acsami.9b12988 31795638

[158] Velmurugan P, Shim J, Kim K, Oh B-T (2016). Prunus × yedoensis tree gum mediated synthesis of platinum nanoparticles with antifungal activity against phytopathogens. Mater Lett.

[159] Osonga FJ, Kalra S, Miller RM, Isika D, Sadik OA (2020). Synthesis, characterization and antifungal activities of eco-friendly palladium nanoparticles. RSC Adv.

[160] Kamli MR, Srivastava V, Hajrah NH, Sabir JSM, Ali A, Malik MA, Ahmad A (2021). Phytogenic fabrication of Ag–Fe bimetallic nanoparticles for cell cycle arrest and apoptosis signaling pathways in Candida auris by generating oxidative stress. Antioxidants.

[161] Kamli MR, Srivastava V, Hajrah NH, Sabir JSM, Hakeem KR, Ahmad A, Malik MA (2021). Facile bio-fabrication of Ag–Cu–Co trimetallic nanoparticles and its fungicidal activity against Candida auris. J Fungi (Basel).

[162] Sivaraj R, Rahman PKSM, Rajiv P, Narendhran S, Venckatesh R (2014). Biosynthesis and characterization of Acalypha indica mediated copper oxide nanoparticles and evaluation of its antimicrobial and anticancer activity. Spectrochim Acta Part A Mol Biomol Spectrosc.

[163] Shenashen M, Derbalah A, Hamza A, Mohamed A, El Safty S (2017). Antifungal activity of fabricated mesoporous alumina nanoparticles against root rot disease of tomato caused by Fusarium oxysporium. Pest Manag Sci.

[164] Parizi MA, Moradpour Y, Roostaei A, Khani M, Negahdari M, Rahimi G (2014). Evaluation of the antifungal effect of magnesium oxide nanoparticles on Fusarium oxysporum F. Sp. lycopersici, pathogenic agent of tomato. Eur J Exp Biol.

[165] Karimiyan A, Najafzadeh H, Ghorbanpoor M, Hekmatimoghaddam S (2015). Antifungal effect of magnesium oxide, zinc oxide, silicon oxide and copper oxide nanoparticles against Candida albicans. Zahedan J Res Med Sci.

[166] Mosleh-Shirazi S, Abbasi M, Moaddeli MR, Vaez A, Shafiee M, Kasaee SR, Amani AM, Hatam S (2022). Nanotechnology advances in the detection and treatment of cancer: an overview. Nanotheranostics.

[167] Haleem A, Javaid M, Singh RP, Rab S, Suman R (2023). Applications of nanotechnology in medical field: a brief review. Glob Health J.

[168] Danaei M, Dehghankhold M, Ataei S, Hasanzadeh Davarani F, Javanmard R, Dokhani A, Khorasani S, Mozafari MR (2018). Impact of particle size and polydispersity index on the clinical applications of lipidic nanocarrier systems. Pharmaceutics.

[169] Leong MY, Kong YL, Burgess K, Wong WF, Sethi G, Looi CY (2023). Recent development of nanomaterials for transdermal drug delivery. Biomedicines.

[170] Simmons R. Research into ancient lineage of microscopic fungi upends assumptions about its genetic relationships. Purude University; 2022. https://www.purdue.edu/newsroom/releases/2022/Q4/research-into-ancient-lineage-of-microscopic-fungi-upends-assumptions-about-its-genetic-relationships.html.

